# Multimodal near‐infrared molecular imaging of ex vivo endometrial carcinoma via CD47‐based targeted tracer

**DOI:** 10.1002/btm2.10754

**Published:** 2025-02-04

**Authors:** Jing Lei, Dianfeng Tian, Bo Zhang, Hongrui Guo, Huancheng Su, Jinzheng Wei, Shuai Li, Sufen Li, Chao Liu, Xiaofeng Yang, Sanyuan Zhang

**Affiliations:** ^1^ Department of Gynecology First Hospital of Shanxi Medical University Taiyuan Shanxi China; ^2^ Department of Coloproctology First Hospital of Shanxi Medical University Taiyuan Shanxi China; ^3^ Department of Breast Surgery Shanxi Province Cancer Hospital Taiyuan Shanxi China; ^4^ Department of Orthopaedics First Hospital of Shanxi Medical University Taiyuan China; ^5^ Department of Urology First Hospital of Shanxi Medical University Taiyuan China; ^6^ Biomedical Engineering Research Center First Hospital of Shanxi Medical University Taiyuan China

**Keywords:** CD47‐Alexa Fluor790, endometrial cancer, multimodal imaging, near‐infrared photoimmunotherapy, optical molecular imaging

## Abstract

The detection and complete eradication of early‐stage small tumors during hysteroscopy remains a significant clinical challenge in preserving fertility for young women with endometrial cancer (EC). The purpose of this study is to verify the feasibility of CD47 as an optical molecular imaging (OMI) target for human EC and to achieve precise localization and identification in hysteroscopic surgery. The results demonstrated that CD47 was overexpressed in EC through bioinformatics, immunohistochemistry, and qRT‐PCR. In EC cell lines, CD47‐targeted near‐infrared photoimmunotherapy (NIR‐PIT) induced cytotoxicity in a light dose‐dependent manner. Laser confocal microscopy revealed that CD47 intervention significantly increased the phagocytic effect of macrophages on EC cells. In the mice model of partial tumor resection mediated by CD47‐targeted OMI, compared to group A (immune therapy alone), group C (NIR‐PIT treatment) mice showed a reduced tumor recurrence rate after NIR‐PIT intervention. However, the difference did not reach statistical significance. We then evaluated the effect of CD47‐targeted NIR‐PIT maintenance therapy on tumor recurrence in mice. The results indicated that, compared to untreated animals, the tumor growth rate was slower in the NIR‐PIT group using CD47‐Alexa Fluor 790 (CD47‐AF790), allowing for more sustained tumor control. The freshly isolated whole uterus specimens from EC patients were co‐incubated with CD47‐AF790, and a significantly enhanced contrast of NIR visible images of tumor tissue was observed, demonstrating high sensitivity and specificity (tumor‐to‐background ratio >5.05). Finally, under fluorescence microscopy, specific fluorescent signals are observed on tumor cells. In conclusion, accurate localization and excision of EC can be accomplished by employing CD47 optical molecular contrast agents with OMI technology. This method shows potential as a viable and promising approach for the precise diagnosis of EC.

**Translational Impact Statement**

In this study, optical molecular imaging targeting CD47 realized precise identification of endometrial cancer lesions, while combined with NIR‐PIT could target the removal of residual lesions and shedding of tumor cells, thus achieving the integrated requirements for tumor diagnosis and treatment. Therefore, the results provided a promising diagnostic and therapeutic method in preserving fertility for young women with endometrial cancer.

## 1 INTRODUCTION

The incidence of endometrial cancer (EC) is showing an increasing trend year by year,^1^ with a growing rate of occurrence among young women of childbearing age.^2^ Women aged less than 40 years account for 3%–14% of EC,^3^ and up to 70% of childbearing‐age patients remain nulliparous at the time of diagnosis.^4^ The fertility‐preserving treatment of EC has become a research focus in the reproductive field of gynecological oncology. However, high‐dose progestin therapy, as recommended by international guidelines for fertility preservation, has an effective rate of about 50%–70%, and the tumor recurrence rate after conservative treatment is 30%–40%.^5^, ^6^ The reason is that the EC lesions in the uterine cavity have not been effectively eradicated and progestin could not effectively suppress the proliferation of cancerous tissues.^7^

In 2010, Mazzon et al. first proposed hysteroscopic resection of lesions followed by progestin therapy as an innovative method of fertility‐preserving treatment for early‐stage EC, which could reduce the recurrence rate and achieve a higher complete remission rate.^8^ However, white‐light hysteroscopy has limitations in identifying tiny and residual tumors and lacks recognition capabilities in nonspecific lesions, leading to missed diagnoses of tumors, which is one of the main reasons for the high recurrence rate of conservative treatment. How to accurately detect and define the boundary of the tumor, and completely remove the tumor while reducing the damage of normal tissues has become an urgent problem to be solved in the fertility‐preserving treatment of EC.

Optical molecular imaging (OMI) is the fusion of new optical imaging technology and modern molecular biology, which combines targeted fluorescent tracers with over‐expressed molecules on tumor cells for precise identification and functional imaging of specific tissues.^9^ In particular, molecular imaging within the near‐infrared (NIR) spectrum (650–900 nm) has shown many clinical advantages, including increased tissue penetration of light, limited autofluorescence, and safety without radiotoxicity.^10^ Near‐infrared photoimmunotherapy (NIR‐PIT) is one such novel molecular‐targeted cancer diagnostic and therapeutic method, which is conjugated antibodies bound to the surface markers of cancer cells with hydrophilic photosensitizers, specifically killing cancer cells under the excitation of NIR light.^11^ Therefore, applying NIR‐PIT to EC for fertility preservation, during hysteroscopic tumor resection, NIR molecular imaging can provide real‐time imaging to evaluate the lesions, and detect tiny and occult tumors, while photoimmunotherapy can specifically kill the residual and exfoliated tumor cells, which can be used as an effective supplement to optimize the conservative treatment of EC.

CD47 is a membrane protein widely expressed in various tumor cells.^12^ Its high expression in tumor cells is a common mechanism to evade immune attack.^13^ CD47 has been reported to be highly expressed in EC,^14^, ^15^ hence CD47 is expected to be a promising target for OMI of EC. In this study, we explored that a CD47‐targeted NIR molecular probe exhibited specific binding ability and direct killing effect on EC cell lines, enabled qualitative tumor identification and reduced the tumor growth rate in the xenograft mice model, and effectively distinguished EC tissues from adjacent normal tissues in vitro. Our study provided an innovative option for the fertility‐preserving treatment of EC.

## 2 MATERIALS AND METHODS

### 2.1 Bioinformatics analysis

#### 2.1.1 The Cancer Genome Atlas database

The clinical information and transcriptomic data of EC were downloaded from the TCGA database (https://cancergenome.nih.gov/). A total of 583 cases of EC data were obtained, including 548 tumor samples and 35 adjacent samples. Differentially expressed genes (DEGs) were analyzed using the DEseq2 package in R 4.3.1. Significant DEGs were identified using a cutoff of log2FC > 1 (fold change FC) and *p* < 0.05.

#### 2.1.2 Gene Expression Profiling Interactive Analysis2 database

The GEPIA2 bioinformatics database (http://gepia2.cancer-pku.cn/) is used to explore the differential expression analysis between tumors and normal tissues, tumor type, or pathological stage from TCGA and GTEx databases. Through GEPIA2, the expression of CD47 in various human cancers and adjacent normal tissues was obtained. Furthermore, the expression of CD47 in EC and corresponding normal tissues was analyzed.

#### 2.1.3 The University of Alabama at Birmingham Cancer data analysis portal (UALCAN)

UALCAN (http://ualcan.path.uab.edu) is an open public web resource for analyzing mRNA expression of potential genes in various tumor subtypes, including age, gender, tumor staging, and other clinicopathological features. In our study, we used UALCAN to analyze the relationship between CD47 expression levels and overall patient survival.

### 2.2 Histology

The paraffin‐embedded pathological specimens of EC and normal endometrial tissues were collected from the First Hospital of Shanxi Medical University after Ethical Committee approval. Patients with EC treated with preoperative radiotherapy and chemotherapy or combined with other malignant tumors were excluded. Cases with complete clinical and follow‐up data were collected, and paraffin‐embedded specimens and clinical data of 53 tumors and 20 contemporaneous normal endometrium from January 2023 to June 2023 were finally included. The tissue blocks were sectioned into 4‐μm slices, mounted on slides, deparaffinized, rehydrated, heat‐antigen repaired, and endogenous peroxidase inactivated. The slices were incubated with a mouse anti‐human CD47 monoclonal antibody (dilution 1:100, sc‐12730, Santa Cruz Biotechnology, Santa Cruz, CA, USA) overnight at 4°C, and then exposed to horseradish peroxidase (HRP)‐labeled IgG (Shanghai Gene Tech) for 30 min. Finally, the antibodies were visualized using diaminobenzidine (DAB) (Shanghai Gene Tech, China) and the slices were counterstained with hematoxylin. The outcomes were assessed using a double‐blind approach, with five non‐overlapping stained fields randomly chosen from each immunohistochemical section at a ×200 magnification. The ImageJ software (National Institutes of Health, Bethesda, MD, USA) was employed to calculate the mean optical density (signal intensity to area ratio) under standardized conditions.

### 2.3 Human EC cell lines

A total of four cell lines were used for the experiments, including human EC cell lines (KLE, HEC‐1‐A, and Ishikawa), and human normal endometrial epithelial cells. KLE and HEC‐1‐A were purchased from Procell Life Science & Technology (Hubei, China). Ishikawa and human normal endometrial epithelial cells were bought from Immocell Biotechnology (Fujian, China). All cells were cultured under the corresponding conditions recommended by the related official website.

### 2.4 Reverse transcription‐quantitative polymerase chain reaction

Total RNA was respectively extracted from KLE, HEC‐1‐A, Ishikawa, and normal endometrial epithelial cells by the TRIzol reagent (Thermo Fisher Scientific). The spectrophotometer (Thermo Fisher Scientific) was used to measure the concentration of total RNA. cDNA was synthesized from appropriate amounts of total RNA using a reverse transcription kit (TransGen Biotech Co., Ltd., Beijing, China). Real‐time PCR was performed using LightCycler480IIReal‐time PCR Instrument (Roche, Swiss) with the SYBR Green Master Mix (ABclonal Technology Co., Ltd., Wuhan, China). The 2 − ΔΔCt method was used to calculate fold changes in the gene expression normalized to GAPDH. The primer sequences were shown as follows: human CD47: 5′‐GAATGCTACTGGCCTTGGTTTA‐3′ (forward) and 5′‐AACCAATATGGCAATGACGAAG‐3′ (reverse) and human GAPDH: 5′‐GGAAGCTTGTCATCAATGGAAATC‐3′ (forward) and 5′‐TGATGACCCTTTTGGCTCCC‐3′ (reverse).

### 2.5 Flow cytometry

EC cells (KLE, Ishikawa, HEC‐1‐A) and normal endometrial epithelial cells were digested with trypsin when both cells had grown to about 80% confluence, and then incubated with CD47‐FITC (200 μg/mL) and IgG1‐FITC (200 μg/mL) for 30 min at 4°C in the dark. For the blocking experiment, the cells were pre‐treated with 1 mg/mL CD47 monoclonal antibody for 10 min, and then incubated with 200 μg/mL CD47‐FITC for 30 min at 4°C. Flow cytometry was performed on the FACSCanto II system (BD Biosciences, USA). The data were analyzed using FlowJo software (version 10.8.1).

### 2.6 Fluorescence microscopy

The cells were placed in 24‐well plates for climbing until the confluence of cells reached about 60%. EC cells (KLE, Ishikawa, HEC‐1‐A) and normal endometrial epithelial cells were washed with PBS, fixed with 4% paraformaldehyde, blocked with 3%BSA, incubated with 1:3000 diluted CD47‐FITC (200 μg/mL) overnight at 4°C in the dark, and then stained with DAPI and mounted. Image acquisition and processing were performed using the Mantra Semi‐automatic Quantitative Pathology Imaging System (Akoya Biosciences, USA). The mean fluorescence intensity (MFI) was calculated using ImageJ software (version 1.53).

### 2.7 In vitro CD47‐targeted NIR‐PIT of EC cell lines

EC cells (KLE, Ishikawa, HEC‐1‐A) were digested with trypsin, washed with PBS, and incubated with CD47‐AF790, CD47, or PBS for 30 min at 4°C in the dark. Cells were then seeded onto 96‐well low‐adhesion plates and irradiated with NIR (760–800 nm) light‐emitting diodes (Shanghai WinWorld Trading, SMO760). The light source was placed at a fixed height of 0.2–0.3 cm above the cell and illuminated with an optical power density of 100 mW/cm^2^. Cells incubated with CD47‐AF790 were exposed to light energy levels of 0, 1, 5, 10, 20, and 40 J/cm^2^, respectively. Cells incubated with CD47 received light energy of 0 and 40 J/cm^2^, respectively. Thirty minutes after cell intervention, 10 μL propidium iodide (PI) 10 μg/mL was added to each well of the 96‐well plate and incubated for 20 min at room temperature. The percentage of cell death was measured on a FACSCanto II flow cytometer system (BD Biosciences, USA). Data were analyzed using FlowJo software (version 10.8.1).

### 2.8 In vitro phagocytosis assay

The phagocytic activity of macrophages on human endometrial carcinoma cell lines in vitro mediated by CD47 antibodies was assessed. EC cells (KLE, Ishikawa, HEC‐1‐A) were digested with trypsin, washed with PBS, and incubated with CD47‐FITC, IgG1‐FITC, or PBS for 30 min at 4°C in the dark. The treated EC cells were added to laser confocal dishes, in which RAW264.7 macrophages were cultured overnight in the laser confocal dishes. The cells were co‐cultured with RAW264.7 macrophages for 2 h at 37°C. The images were observed by laser confocal microscopy (Leica, Germany).

RAW264.7 macrophages were seeded into 96‐well plates and pre‐cultured overnight. KLE cells were digested using trypsin, washed with PBS, and incubated with anti‐CD47‐AF790, anti‐CD47, or PBS for 30 min at 4°C in the dark. The treated KLE cells were co‐cultured with macrophages in 96 Wells. The cells were divided into a 5 J/cm^2^ NIR light group and a non‐irradiation group. Cell viability was measured by CCK8 assay. Absorbance at 450 nm was measured for each well using a microplate reader.

### 2.9 In vivo NIR‐PIT of human EC xenografts

Female athymic NU/NU nude mice (4 weeks old) were purchased from Beijing Vital River Laboratory Animal Technology (Beijing, China), housed under standard pathogen‐free environment laboratory and cared for in accordance with institutional animal care guidelines. All animal experiments were conducted in compliance with the guidelines formulates by the Ethics Committee for Animal Experimentation of Shanxi Medical University (permission: NO. DWYJ‐2024‐298). For all procedures, animals were anesthetized with isoflurane (RWD Life Science Co., Ltd., Shenzhen, China). A total of 3 × 10^6^ KLE cells suspended in 50% Matrigel (Corning) were inoculated subcutaneously into the right forelimb pit of NU/NU nude mice. Tumor resection was conducted when the tumor volume was up to 500 mm^3^. Tumor volume = (length × width^2^)/2.

To evaluate the effectiveness of CD47‐targeted OMI in assisting tumor removal and the therapeutic effects of NIR‐PIT in vivo, mice were randomly divided into three groups, with 10 mice in each group. Anti‐CD47‐AF790 (100 μg) was injected via the tail vein 24 h before tumor resection. Under the guidance of CD47‐targeted OMI, 95% of the tumor were resected in Groups A and C, and the tumor were completely excised in Group B. Mice in group C were given NIR light irradiation of 100 J/cm^2^ immediately and 24 h after surgery.

Prior to wound closure, two independent gynecologists were required to distinguish which mice received complete tumor excision versus partial tumor excision. They had no prior information about the design of the experiment. After adequate hemostasis, the two gynecologists independently inspected the wound without any enhanced visual tools and recorded any suspicious areas.

To confirm the size and location of residual lesions after surgery, micro computed axial tomography (micro CT) (Bruker, Germany) was performed on 15 mice (5 in each group) both before surgery and immediately after surgery but prior to wound closure.

### 2.10 In vivo NIR‐PIT on tumor recurrence in human EC xenografts

To evaluate the effect of CD47‐targeted NIR‐PIT on tumor recurrence in xenograft mouse model, mice with tumor recurrence in group A were randomly divided into 2 groups: (1) control group without any intervention; (2) NIR‐PIT group, which received an intravenous injection of 100 μg of anti‐CD47‐AF790 in the first week, followed by NIR irradiation at a dose of 100 J/cm^2^ on day 1 and day 2, once a week for 3 weeks as described above.

### 2.11 Ex vivo OMI of tumor tissue

Three nude mice were randomly selected from groups B and C, and tissue samples (muscle, fat, and skin) were collected 0.5 cm from the tumor margins. Using a near‐infrared imaging system, the fluorescence signal intensity of the tumor tissue and normal tissues (muscle, fat, and skin) was measured, and the average fluorescence grayscale values were calculated.

### 2.12 Ex vivo near‐infrared molecular imaging

With the approval of our institution's ethics committee, we prospectively enrolled 16 patients with EC from February 2023 to April 2024. The participants included in the study were diagnosed with primary, isolated, and non‐recurring endometrial adenocarcinoma through diagnostic curettage pathology, transvaginal ultrasound, and pelvic MRI. We excluded individuals with possible concurrent ovarian cancer, preoperative indications of distant metastasis, or tumors exceeding 5 cm. All patients underwent a laparoscopic total hysterectomy with bilateral salpingo‐oophorectomy and surgical staging. Additionally, all participants were given written consent and signed the informed consent form for the study.

**FIGURE 1:**
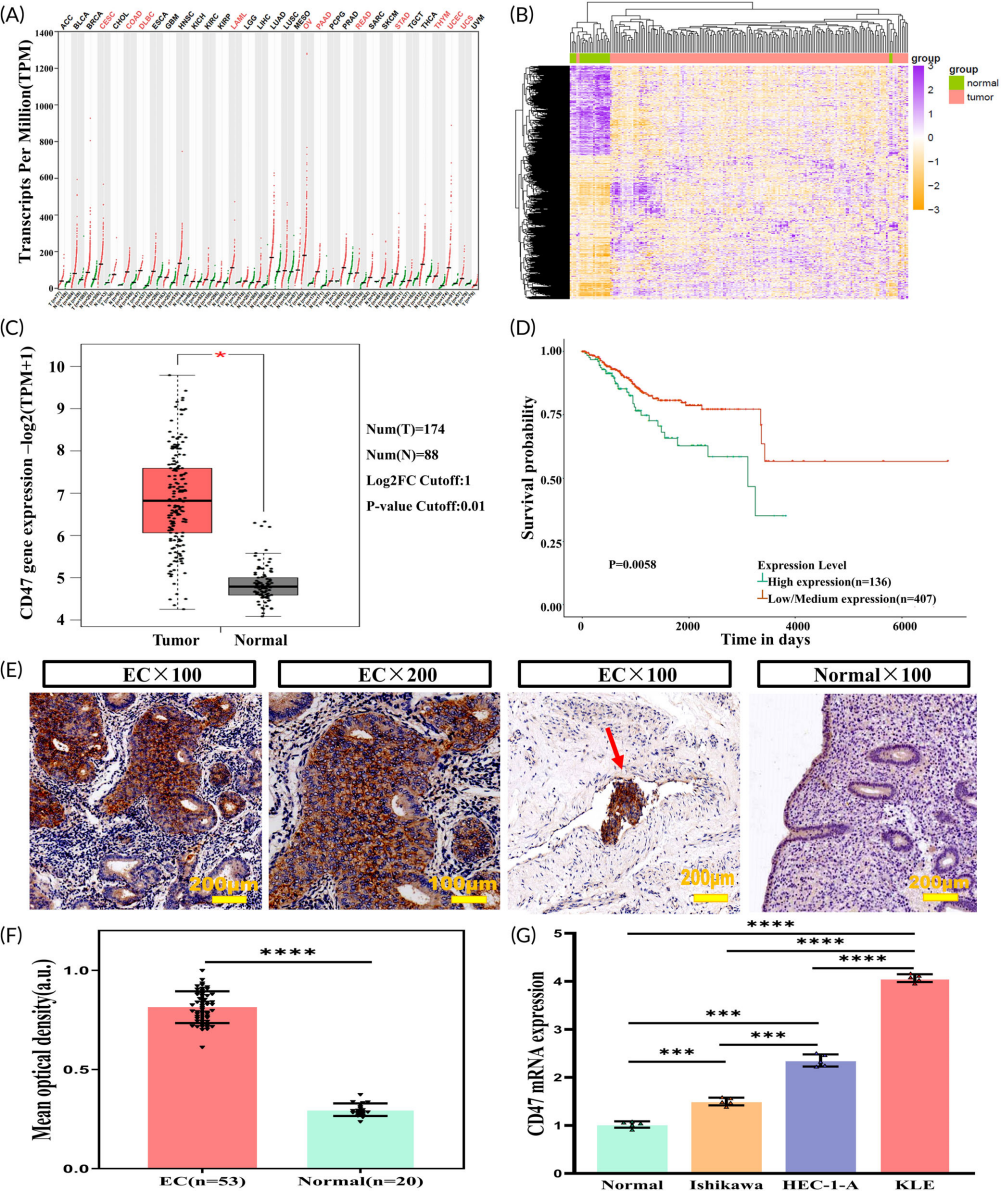
CD47 is overexpressed in EC. (A) CD47 expression of all tumor (red) and normal (green) tissues from GEPIA2 databases. (B) Hierarchical clustering of DEGs, with rows representing genes and columns representing samples. For a gene, purple represents a higher expression level, orange represents a lower expression level, and white represents the median expression level for all samples. (C) The expression of CD47 mRNA in EC tissues (red box) and paired normal tissues (black box) from GEPIA2. (D) Kaplan–Meier curves based on CD47 expression for UCEC patients from UALCAN. (E) Representative IHC staining for CD47 in EC and normal endometrium. The red arrow indicates CD47‐specific labeling of EC embolus in the vasculature. (F) Qualitative analysis of IHC results revealed that CD47 protein expression was higher in EC (*n* = 53) than in normal tissue (*n* = 20) (*p* < 0.0001). (G) The expression of CD47 mRNA in EC cells compared to normal endometrial epithelial cells.

Fresh and intact whole uterine specimens were collected from the operating room immediately after surgical resection. Anti‐CD47‐Alexa Fluor 790 (excitation, 760 nm; emission, 835 nm; 200 mg/mL; Santa Cruz Biotechnology, Santa Cruz, CA, USA) were diluted in phosphate‐buffered saline at a 1:100 ratio to prepare 7 mL of imaging targeting agent and slowly injected into the uterine cavity through a 12B fallopian tubal catheter. After incubation at 37°C for 30 min, the specimen was rinsed three times with sterile saline to remove unbound antibodies. The cleaned whole uterine specimen was then Y‐dissected through the anterior uterine wall and placed under a Visible Light and Near‐Infrared Fluorescence Separation Combined Imaging System (SES Co., Taiyuan, China) for imaging. This imaging system captures real‐time visible light and NIR fluorescence information from the uterine specimen. Subsequently, white light images, fluorescence images, and fused images are displayed simultaneously on the screen (Figure 1).

Positive anti‐CD47‐Alexa Fluor 790 fluorescence was defined as a bright area in the NIR grayscale images that was independent of the shooting angle. The MFI of the corresponding tissue was expressed according to the average grayscale values of the tumor and the adjacent normal background in the NIR grayscale image. Tumor to background ratio (TBR) was calculated as the ratio of MFI of tumor tissue to MFI of normal background tissue. After imaging, fluorescent areas were marked with ink and submitted in full to the same pathologist for standard histopathology, according to the 2023 FIGO clinical practice guidelines for EC.

### 2.13 Immunofluorescence

The fluorescent tumor tissues and non‐fluorescent normal tissues adjacent to the tumor from ex vivo NIR imaging specimens were sectioned in a cryostat at 4 μm for hematoxylin and eosin (H&E) staining and fluorescence microscopy examination, respectively. The frozen sections were subsequently stained with 4′,6‐diamidino‐2‐phenylindole (DAPI, Boster, China) to highlight the cell nuclei. After covering with coverslips, samples were visualized using the Mantra semi‐automated quantitative Pathology Imaging system (Akoya Biosciences, USA) for image capture and processing. Adjacent sections were stained with hematoxylin and eosin (H&E) to confirm the presence of malignant lesions.

### 2.14 Statistical analysis

Quantitative data adhering to normal distribution were expressed as mean ± standard deviation, and their differences were evaluated using the *t*‐test. For measurements not following normal distribution, values were represented as median (range) and assessed using the Wilcoxon rank‐sum test. Qualitative data were illustrated as frequencies and percentages, with comparisons made using either the chi‐square test or Fisher's exact test. For fluorescence intensity of in vitro tissue specimens, an unpaired *t*‐test was used to assess the difference between the two groups. Analyses were performed using R 4.3.1 and GraphPad Prism 9.5, recognizing a *p*‐value <0.05 to signify statistical significance. All statistical examinations were two‐tailed.

## 3 RESULTS

### 3.1 CD47 is overexpressed in human EC

By reviewing the published literature and preliminary experimental data, we have observed differential expression of CD47 mRNA in EC. Therefore, we believe that CD47 may be a viable OMI target for endometrial carcinoma. We first identified the expression profile of CD47 using GEPIA2, which showed significant upregulation of CD47 in several cancer types including cervical squamous cell carcinoma (CESC), colon adenocarcinoma (COAD), diffuse large B‐cell lymphoma (DLBC), acute myeloid leukemia (LAML), ovarian carcinoma (OV), pancreatic adenocarcinoma (PAAD), rectum adenocarcinoma (READ), stomach adenocarcinoma (STAD), thymoma (THYM), uterine corpus endometrial carcinoma (UCEC), and uterine carcinosarcoma (UCS) (*p* < 0.05, Figure 1A). The results revealed that EC is one of the tumors with elevated expression in the CD47 expression profile. Then we extracted EC transcriptome data from the TCGA database, encompassing 548 EC specimens and 35 normal adjacent samples. This data was processed using R 4.3.1, which included normalization, log transformation, and the removal of unannotated and duplicate entries. A total of 39,332 genes were screened out, of which 9184 genes were up‐regulated and 4255 genes were down‐regulated (Figure 1B). We found CD47 is one of the upregulated genes in EC. To verify CD47's expression in EC, we discovered a significantly higher transcription level of CD47 in EC tissues compared to normal tissues using GEPIA2 (*p* < 0.05, Figure 1C). Additionally, analysis using the Kaplan–Meier method from the UALCAN database showed that patients with high expression of CD47 had poorer overall survival (*p* < 0.01, Figure 1D).

CD47 expression was validated by immunohistochemical analysis of 53 EC and 20 normal endometrial tissues from our institution. Detailed clinical and pathological data of these patients are shown in Table 1. The results demonstrated that CD47 expression in EC (*n* = 53) was significantly elevated compared to normal endometrial tissues (*n* = 20), with mean optical densities of 0.8124 ± 0.0802 and 0.2971 ± 0.0318, respectively (*p* < 0.0001, Figure 1F). Overall, these data indicated that CD47 could become a potential imaging target for precise identification of EC.

**TABLE 1:** Clinical and pathologic information of patients with immunohistochemical staining.

Variable	EC (*n* = 53)
Age (years)	55.4 (33–74)
Tumor diameter (cm)	2.9 (0.5–10)
FIGO stage, *n* (%)	
I stage	46 (86.8%)
III–IV stage	7 (13.2%)
Pathology grade	
G1	22 (41.5%)
G2	23 (43.4%)
G3	8 (15.1%)
Pathology subtype	
Endometrioid	51 (96.2%)
Serous carcinoma	1 (1.9%)
Undifferentiated carcinoma	1 (1.9%)

*Note*: FIGO, The International Federation of Gynecology and Obstetrics; G1, low grade; G2, middle grade; G3, high grade.

The expression of CD47 mRNA in EC cell lines (Ishikawa, HEC‐1‐A, KLE), and normal human endometrial epithelial cells was assessed using qRT‐PCR. Compared to normal human endometrial epithelial cells, the expression levels of CD47 mRNA were significantly higher in EC cell lines (Ishikawa vs. normal, *p* = 0.0008; HEC‐1‐A vs. normal, *p* = 0.0001; KLE vs. normal, *p* < 0.0001) (Figure 1G), and among the endometrial cell lines, KLE showed the highest expression levels (KLE vs. Ishikawa, *p* < 0.0001; KLE vs. HEC‐1‐A, *p* < 0.0001). This further validated the feasibility of CD47 as a target for OMI in human EC.

### 3.2 CD47 targeting enhanced cell binding capacity

**FIGURE 2:**
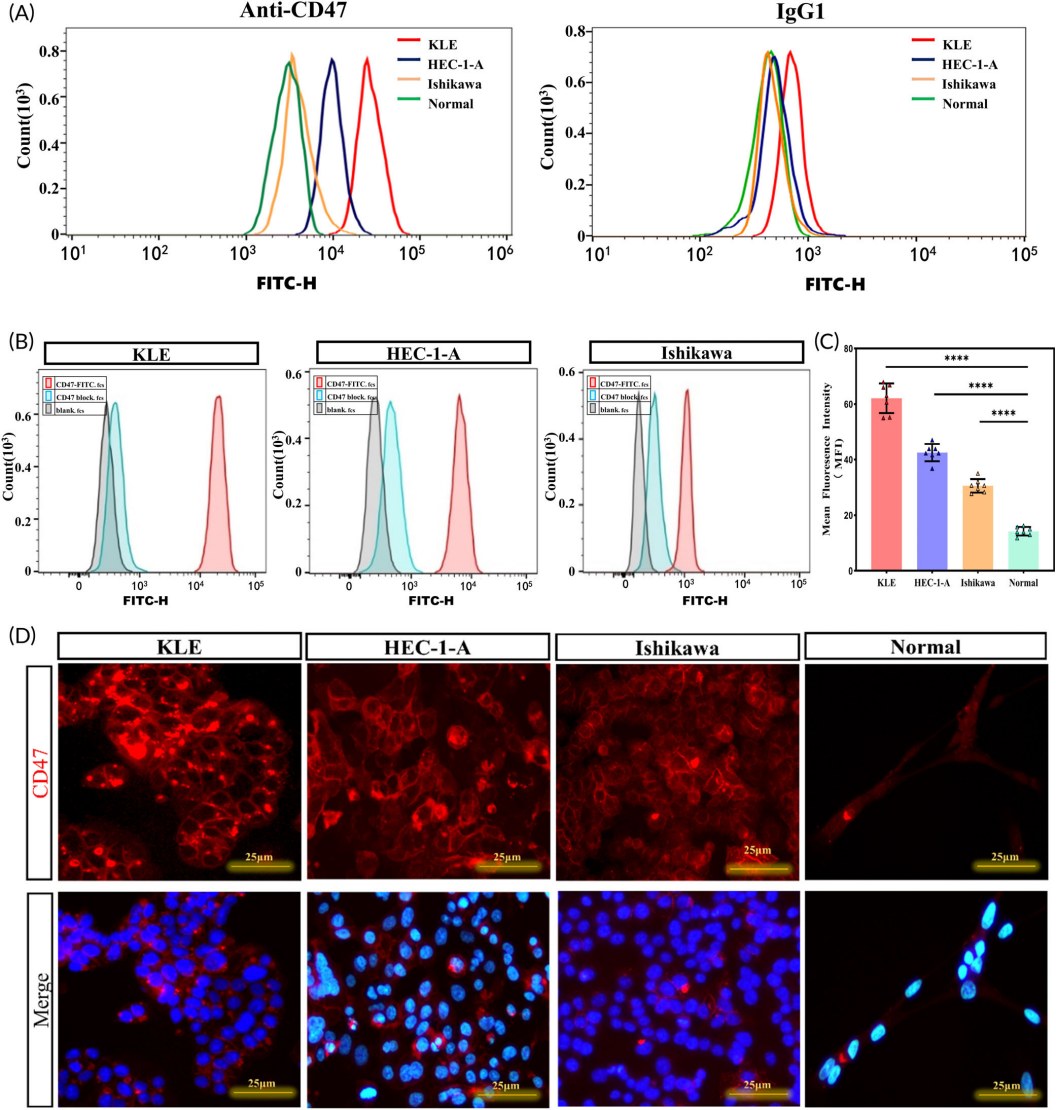
Cell binding study of CD47. (A) Differences in the affinity of CD47 antibodies and its isotype control antibody IgG1 with EC cell and normal endometrial epithelial cells. (B) Flow cytometry analysis of EC cells after incubation with CD47‐FITC and CD47. (C) Comparison of mean fluorescence intensity of CD47 between EC cells and normal endometrial epithelial cells in immunofluorescence (****p < 0.0001). (D) Representative fluorescence microscopy images of EC cells after incubation with CD47‐AF790.

To evaluate the difference in the affinity of CD47 antibody between EC cells and human normal endometrial epithelial cells, EC cells or human normal endometrial epithelial cells were incubated with anti‐CD47‐FITC and its isotype control antibody IgG1‐FITC, respectively, and then detected by flow cytometry. Flow cytometry confirmed that the affinity between CD47 and EC cells was significantly higher compared with normal endometrial epithelial cells. IgG1, as an isotype control antibody, showed non‐specific binding to both cancer and normal epithelial cells (Figure 2A). Additionally, in the CD47 blocking experiments of three EC cells, competition of CD47‐FITC with unlabeled CD47 led to a significant reduction in fluorescence intensity (Figure 2B).

In addition, to test the specific binding ability and site of anti‐CD47‐AF790 with EC cells, an immunofluorescence assay revealed that the fluorescence intensity of EC cells was significantly enhanced compared with normal human endometrial epithelial cells, and mainly concentrated on the tumor cell membrane (Figure 2C,D). Taken together, these results demonstrate the CD47‐specific binding ability to EC.

### 3.3 In vitro NIR‐PIT of human EC cell lines

To evaluate the direct cytotoxicity following NIR‐PIT via anti‐CD47‐AF790, three human EC cell lines (KLE, HEC‐1‐A, and Ishikawa) were incubated with anti‐CD47‐AF790 and exposed to increasing doses of NIR light (0–40 J/cm^2^). The proportion of dead cells was assessed by flow cytometry using propidium iodide.

**FIGURE 3:**
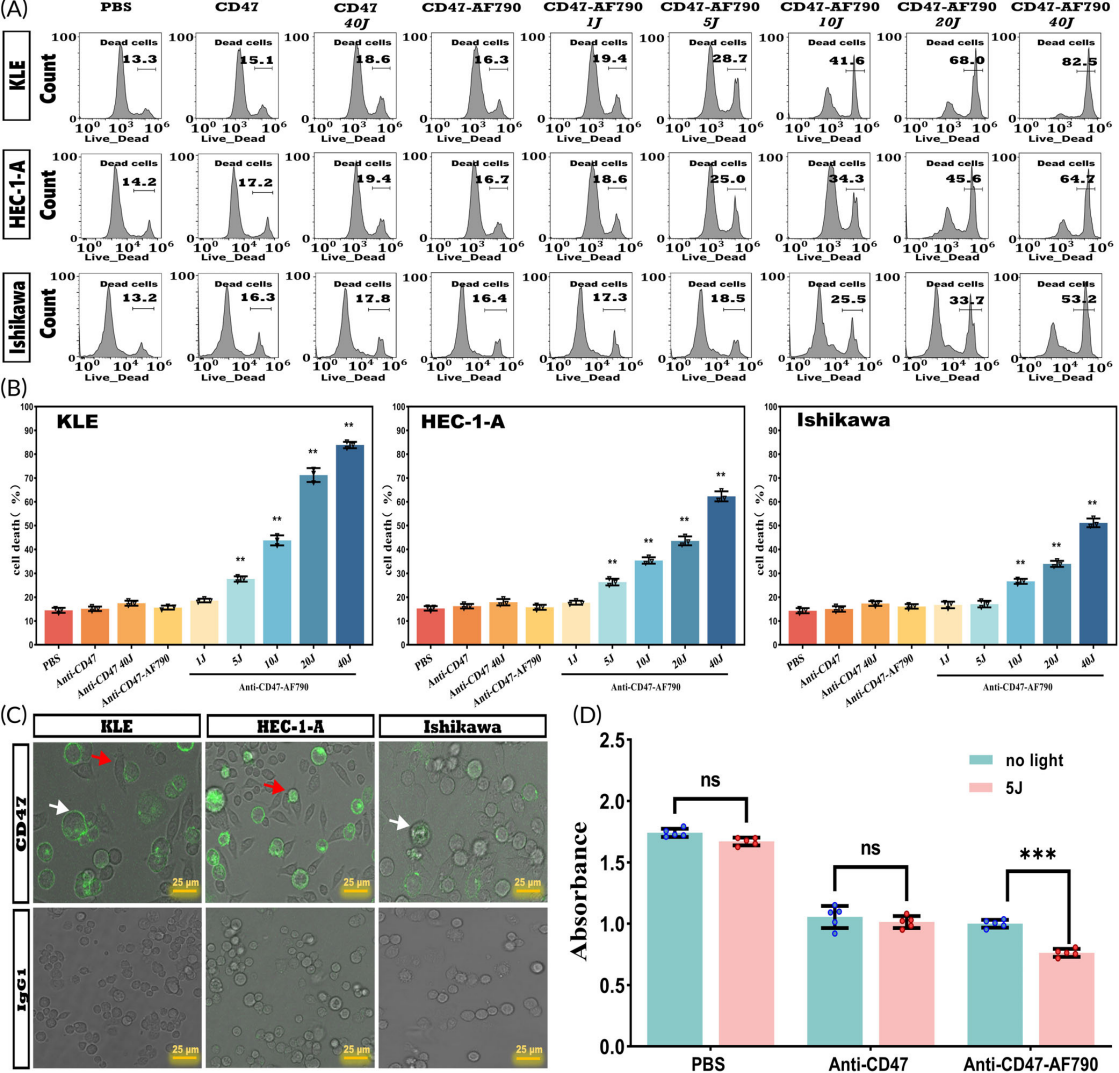
In vitro CD47‐targeted NIR‐PIT for EC cell lines. (A) In EC cell lines, the percentages of cell death with different light doses were mediated by NIR‐PIT. (B) In EC cell lines, CD47‐targeted NIR‐PIT led to increased cell death in a light‐dose‐dependent manner. Significant cancer cell death started at 5 J/cm^2^ in KLE and HEC‐1‐A cells, and 10 J/cm^2^ in Ishikawa cells, and the percentages of cell death increased with the increase of NIR light energy. At the maximum irradiation dose of 40 J/cm^2^, more than 80% of KLE cells and over 50% of HEC‐1‐A and Ishikawa cells died. (C) The phagocytosis of macrophages by laser confocal microscopy. The white arrow indicated that tumor cells were phagocytized by macrophages; the red arrow displayed that macrophages were chemotactic and deformed to phagocytize tumor cells. (D) KLE cells were incubated with PBS, CD47 antibody, or CD47‐AF790 and then seeded onto 96‐well plates pre‐cultured with RAW 264.7 macrophages. Compared with the control group without light treatment, the absorbance values of the cells incubated with CD47‐AF790 significantly decreased under 5 J/cm^2^ light exposure (*p* < 0.001), but there was no significant change in the absorbance values between the PBS group and the CD47 group (*p* = 0.17, *p* = 0.58).

Under NIR‐PIT intervention, the death of EC cells was increased in a light dose‐dependent manner (Figure 3A). The percentages of cell death began to rise at a light dosage of 5 J/cm^2^ for KLE and HEC‐1‐A cells, and at 10 J/cm^2^ for Ishikawa cells. At the maximum irradiation dose of 40 J/cm^2^, more than 80% of KLE cells and over 50% of HEC‐1‐A and Ishikawa cells died (Figure 3B). To control the effect of NIR alone, the three EC cells were incubated with unlabeled anti‐CD47, and the percentages of cell death was observed under the intervention of no light or maximum dose (40 J/cm^2^) NIR light. Compared with the PBS control group, there was no statistically significant difference in the percentage of cell death in the three EC cell lines under no light or maximum dose of NIR light.

To confirm that CD47 antibodies could block the immune evasion attack of cancer cells in macrophages,^13^ the EC lines (KLE, HEC‐1‐A, and Ishikawa) were incubated with anti‐CD47‐FITC and then co‐cultured with macrophages, and the phagocytosis of macrophages was observed by laser confocal microscopy. The intervention with CD47 antibodies significantly increased the phagocytosis of cancer cells by macrophages compared with cancer cell lines treated with isotype control antibody IgG1 (Figure 3C).

KLE cells exhibited the highest CD47 mRNA expression levels among the three EC cell lines, so our study explored the CD47‐mediated phagocytosis of KLE cells by macrophages in vitro under NIR‐PIT. KLE cells were incubated with PBS, CD47 antibody, or CD47‐AF790, and incubated KLE cells were seeded onto 96‐well plates pre‐cultured with RAW 264.7 macrophages. Compared with the control group without light treatment, the absorbance values of the cells incubated with CD47‐AF790 significantly decreased under 5 J/cm^2^ light exposure (*p* < 0.001). However, there was no significant change in the absorbance values between the PBS group and the anti‐CD47 group (*p* = 0.17, *p* = 0.58, Figure 3D). The experiment demonstrated that anti‐CD47‐AF790 has a dual function of directly inducing cell death and enhancing phagocytosis through NIR‐PIT mediation.

### 3.4 Development of local relapses in surgical models with residual tumor after surgery

To evaluate the intraoperative real‐time guidance provided by anti‐CD47 targeted probe‐mediated OMI, partial or complete tumor resections were performed in EC xenograft models. Two gynecologists were asked to independently assess whether residual tumor after visual inspection at the time of wound opening. The gynecologists could only detect that 2 of 20 mice with partially excised tumors (groups A and C) but incorrectly identify that 2 of 10 mice with completely excised tumors (groups B) had residual tumors. Consequently, the sensitivity, specificity, positive predictive value and negative predictive value of visual inspection of the wound for residual tumors were 10% (2/20), 80% (8/10), 50% (2/4), and 30.8% (8/26), respectively.

**FIGURE 4:**
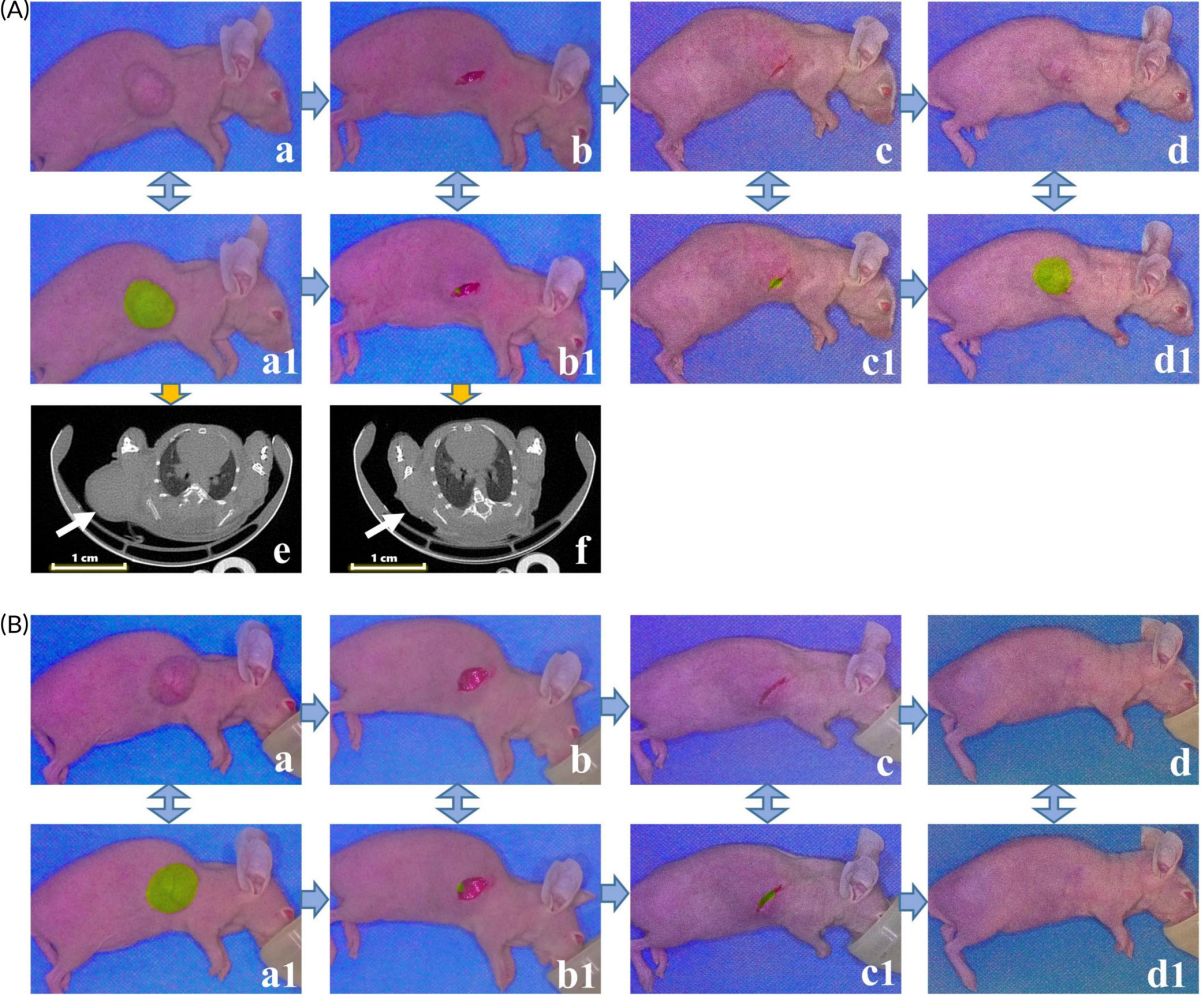
CD47‐targeted optical molecular imaging in a mouse model of residual tumor. (A) Tumor recurrence in mice of residual tumor after NIR‐PIT; (a) visible light image of the xenograft mouse model; (b) visible light image of the residual tumor before wound closure; (c) visible light image of mice with residual tumors after receiving NIR‐PIT at 24 h postoperatively; (d) visible light image of mice with recurrent tumor; (a1–d1) fusion image of visible light and fluorescence corresponding to a–d image under optical molecular imaging; (e) micro CT image of the subcutaneous EC xenograft mouse; (f) micro CT image of the residual tumor after tumor resection (the white arrows indicated the tumor location before and after resection). (B) No tumor recurrence in mice of residual tumor after NIR‐PIT; (a) visible light image of the xenograft mouse model; (b) visible light image of the residual tumor before wound closure; (c) visible light image of mice with residual tumors after receiving NIR‐PIT at 24 h postoperatively; (d) visible light image of mice with no tumor recurrence; (a1–d1) fusion image of visible light and fluorescence corresponding to a–d image under optical molecular imaging.

Micro CT scanning of tumor volumes in subcutaneous mouse xenograft were more accurate than external caliper measurements.^16^ Therefore, we performed radiographic imaging on the surgical wounds of the nude mice to assess for residual tumors. The results indicated that micro CT scanning was unable to discern the residual tumors from surrounding normal tissue (Figure 4A.e,f).

Partial resection of the tumor was conducted in group A and group C. The postoperative tumor recurrence rate was 70% (7/10) in group A. In group C, where mice received NIR‐PIT treatment with 100 J/cm^2^ NIR light irradiation immediately after surgery and again 24 h later, the recurrence rate was 50% (5/10) (Figure 4A), and 50% (5/10) mice did not have tumor recurrence (Figure 4B). Compared with the tumor recurrence rate in group A, the recurrence rate in group C showed a decreasing trend, but the difference was not statistically significant (*p* = 0.65). Complete resection of the tumor was performed in group B, and no obvious tumor recurrence was observed after the operation.

### 3.5 Therapeutic effect of CD47‐targeted NIR‐PIT on tumor recurrence in xenograft mouse model

**FIGURE 5:**
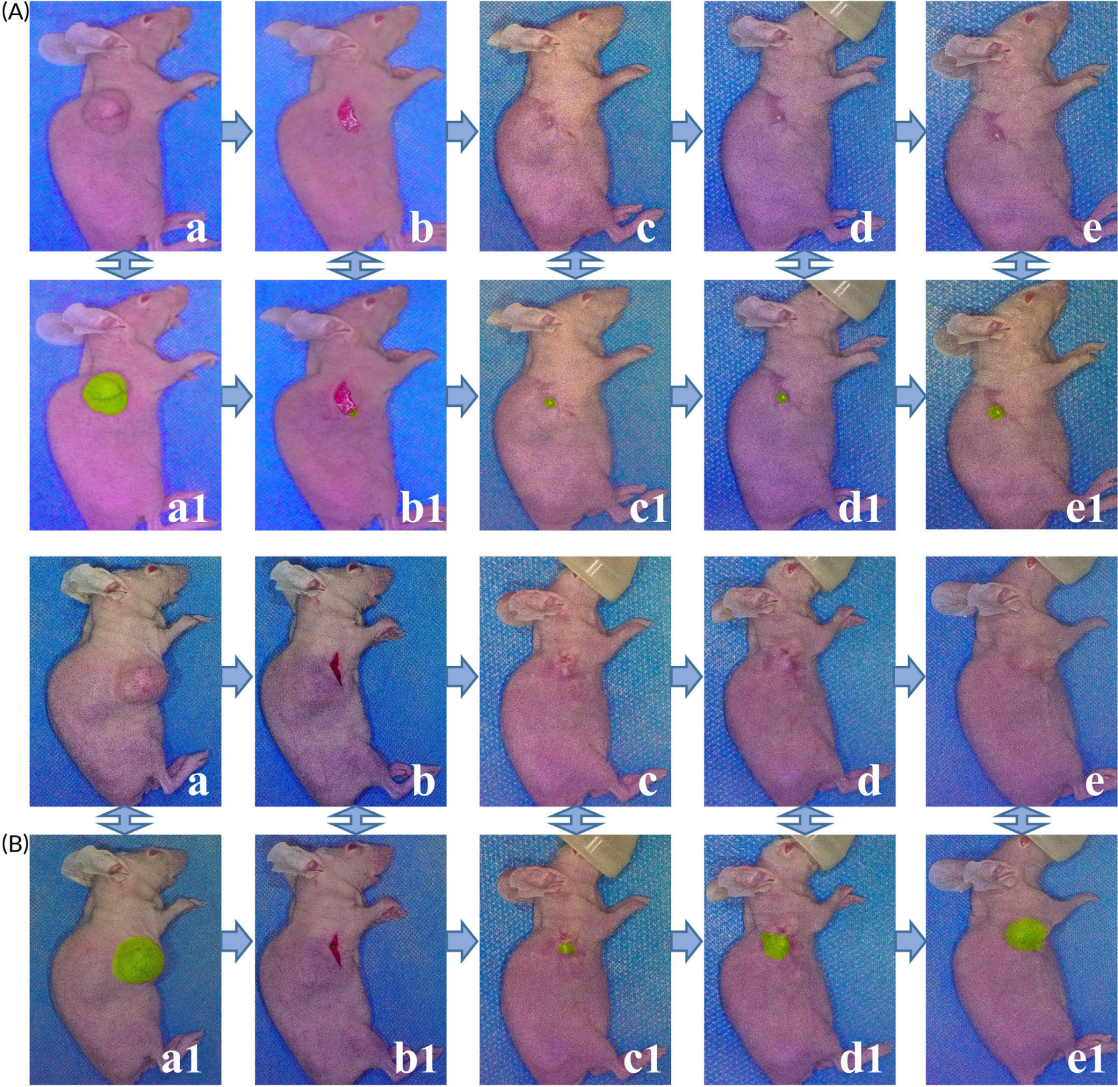
Therapeutic response of CD47‐targeted NIR‐PIT on tumor recurrence in xenograft mouse model. (A) Representative animals showed a reduced tumor growth rate under CD47‐targeted NIR‐PIT; (a) visible light image of the xenograft mouse model; (b) visible light image of mice with residual tumor before wound closure; (c) visible light image of mice with recurrent tumor after NIR‐PIT treatment at the first week after surgery; (d) visible light image of mice with recurrent tumor after NIR‐PIT treatment at the second week after surgery; (e) visible light image of mice with recurrent tumor after NIR‐PIT treatment at the third week after surgery; (a1–e1) Fusion image of visible light and fluorescence corresponding to a–d image under optical molecular imaging. (B) Representative animals in the control group exhibited significantly larger recurrent tumor growth; (a) visible light image of the xenograft mouse model; (b) visible light image of mice with residual tumor before wound closure; (c) visible light image of mice with recurrent tumor after NIR‐PIT treatment at the first week after surgery; (d) visible light image of mice with recurrent tumor after NIR‐PIT treatment at the second week after surgery; (e) visible light image of mice with recurrent tumor after NIR‐PIT treatment at the third week after surgery; (a1–e1) fusion image of visible light and fluorescence corresponding to a–d image under optical molecular imaging.

**FIGURE 6:**
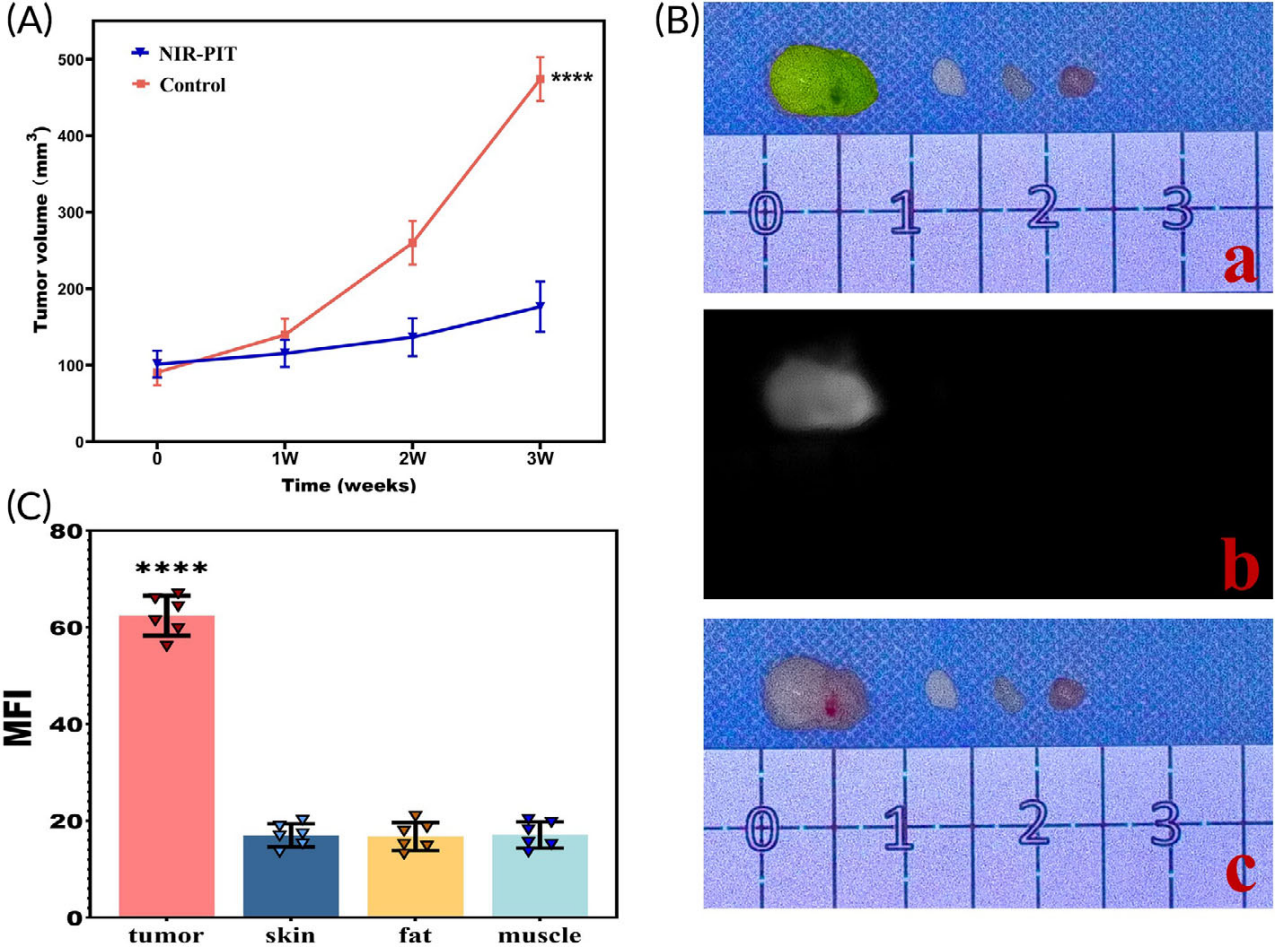
Response of CD47‐targeted NIR‐PIT in recurrent tumor and ex vivo optical molecular imaging of resected tumor tissue and adjacent normal tissues. (A) Compared to the control group, the tumor growth rate in the NIR‐PIT group was significantly reduced (*p* < 0.0001). (B) Visible light (a), fluorescence (b) and merged (c) images of tumor tissue and adjacent normal tissues (skin, fat, and muscle) (from left to right: Tissues are tumor, skin, fat, and muscle). (C) The grayscale values of MFI for tumor tissue and skin, fat, and muscle were 62.41 ± 4.11, 17.01 ± 2.39, 16.76 ± 2.89, and 17.09 ± 2.71, respectively. The MFI grayscale value of the tumor tissue was significantly higher compared to skin, fat, and muscle (*p* < 0.0001). The MFI of tumor tissue was approximately 3.6 times higher than that of the adjacent normal tissues.

To assess the therapeutic effect of CD47‐targeted NIR‐PIT on recurrent tumors in xenograft mouse model, six of the seven mice with recurrent tumors in group A were randomly selected and divided into the NIR‐PIT group (Figure 5A) and the control group (Figure 5B), with three mice in each group. Compared to the control group, the NIR‐PIT group exhibited a significantly lower tumor growth rate (*p* < 0.0001, Figure 6A).

### 3.6 Ex vivo OMI of resected tumor specimens

To further explore the application value of OMI in real‐time surgical guidance, the mean fluorescence signal intensity (MFI) of tumor tissues and adjacent normal tissues (skin, fat, and muscle) were compared in vitro (Figure 6B). The results showed that the MFI grayscale values for the tumor, skin, fat, and muscle were 62.41 ± 4.11, 17.01 ± 2.39, 16.76 ± 2.89, and 17.09 ± 2.71, respectively. The fluorescence signal intensity of the tumor tissue was significantly higher than that of the adjacent normal tissues (*p* < 0.0001), and the difference was statistically significant (Figure 6C). The average MFI of the tumor specimens was approximately 3.6 times higher than that of the adjacent normal tissues.

### 3.7 Ex vivo CD47‐targeted NIR molecular imaging of fresh EC specimens

This study involves 16 patients who underwent laparoscopic staging surgery for EC between January 2023 and April 2024. Their average age was 45.94 years, ranging from 37 to 51 years. Table 2 summarizes the demographic characteristics, histopathological diagnoses, and molecular imaging results of these patients. No adverse morphological changes were observed in the specimens after incubation with CD47‐AF790. The application of CD47‐AF790 did not affect the pathological assessment of EC lesions, nor did it cause any harm or degenerative changes in non‐tumor tissues. Histopathological analysis confirmed that all samples were afflicted with endometrioid endometrial carcinoma (EEC).

**TABLE 2:** Patient's demographic data, histopathological diagnosis and molecular imaging results with anti‐CD47‐Alexa Fluor 790 NIR molecular imaging.

Case no	Age (years)	Tumor diameter (cm)	Pathological diagnosis	Grade	FIGO stage	Tumor MFI/Background MFI	TBR
1	46	0.5	Endometrioid adenocarcinoma	G2	IA	110.29/15.71	7.02
2	50	1.5	Endometrioid adenocarcinoma	G2	IA	113.57/17.71	6.41
3	44	1.2	Endometrioid adenocarcinoma	G1	IA	100.86/13.57	7.43
4	49	1.5	Endometrioid adenocarcinoma	G2	IB	111.71/15.43	7.24
5	46	4	Endometrioid adenocarcinoma	G1	IB	105.86/16.14	6.56
6	47	1.5	Endometrioid adenocarcinoma	G1	IA	117.71/18.86	6.24
7	43	2.5	Endometrioid adenocarcinoma	G1	IB	115.57/13.86	8.34
8	45	3	Endometrioid adenocarcinoma	G2	IA	118.86/15.71	7.56
9	48	4	Endometrioid adenocarcinoma	G3	IB	121.57/21.29	5.71
10	46	1.5	Endometrioid adenocarcinoma	G2	IIIC1	123.29/24.43	5.05
11	51	0.5	Endometrioid adenocarcinoma	G2	IA	113.14/16.57	6.82
12	49	0.5	Endometrioid adenocarcinoma	G1	IA	106.86/13.57	7.87
13	37	4	Endometrioid adenocarcinoma	G2	IA	105.29/15.43	6.82
14	51	2.5	Endometrioid adenocarcinoma	G1	IA	103.14/12.43	8.30
15	43	3.5	Endometrioid adenocarcinoma	G2	IB	123.43/16.43	7.51
16	40	3.0	Endometrioid adenocarcinoma	G1	IA	104.43/15.14	6.89

*Note*: FIGO, The International Federation of Gynecology and Obstetrics; G1, low grade; G2, middle grade; G3, high grade.

**FIGURE 7:**
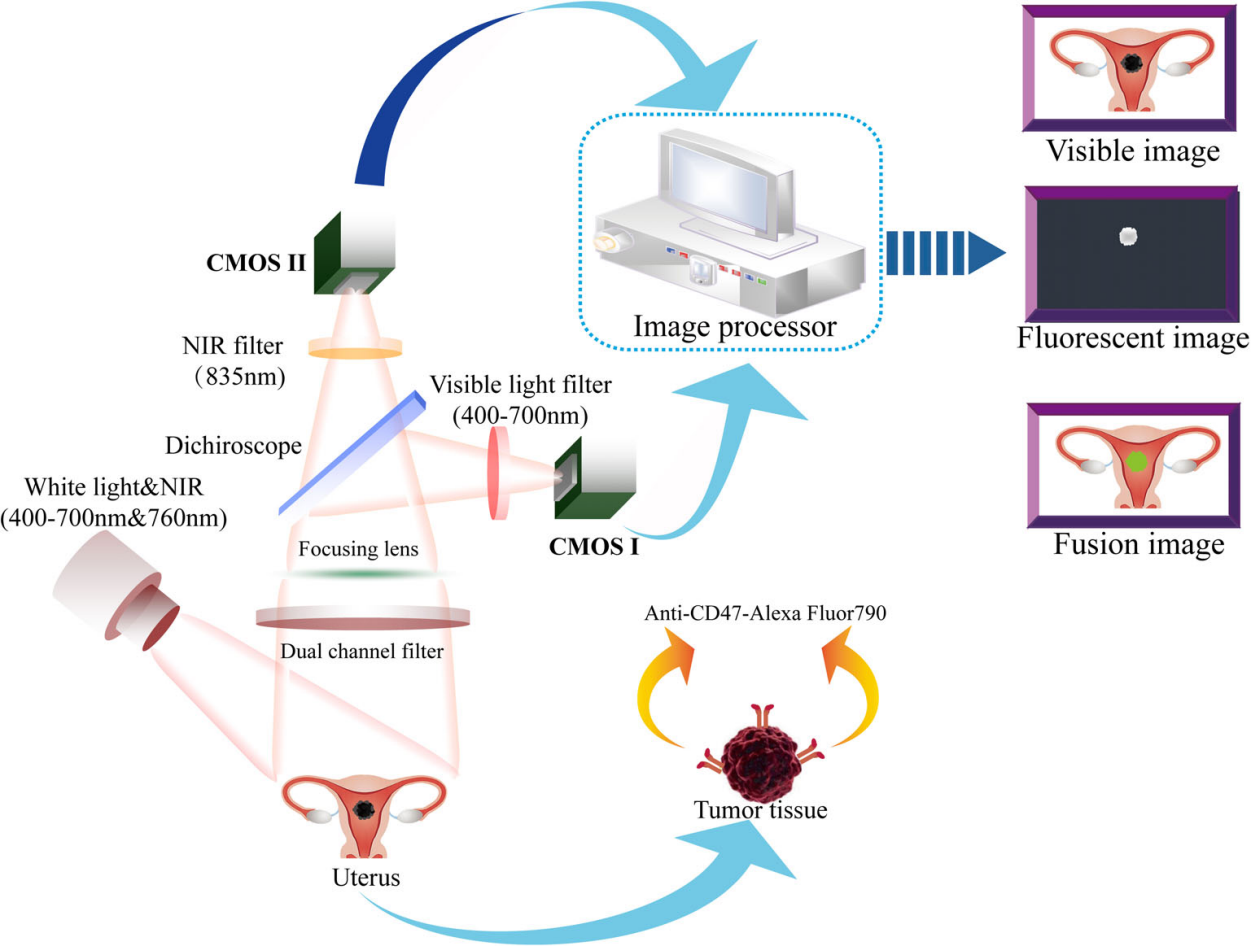
Schematic diagram of the NIR molecular imaging system. CMOS, complementary metal oxide semiconductor.

**FIGURE 8:**
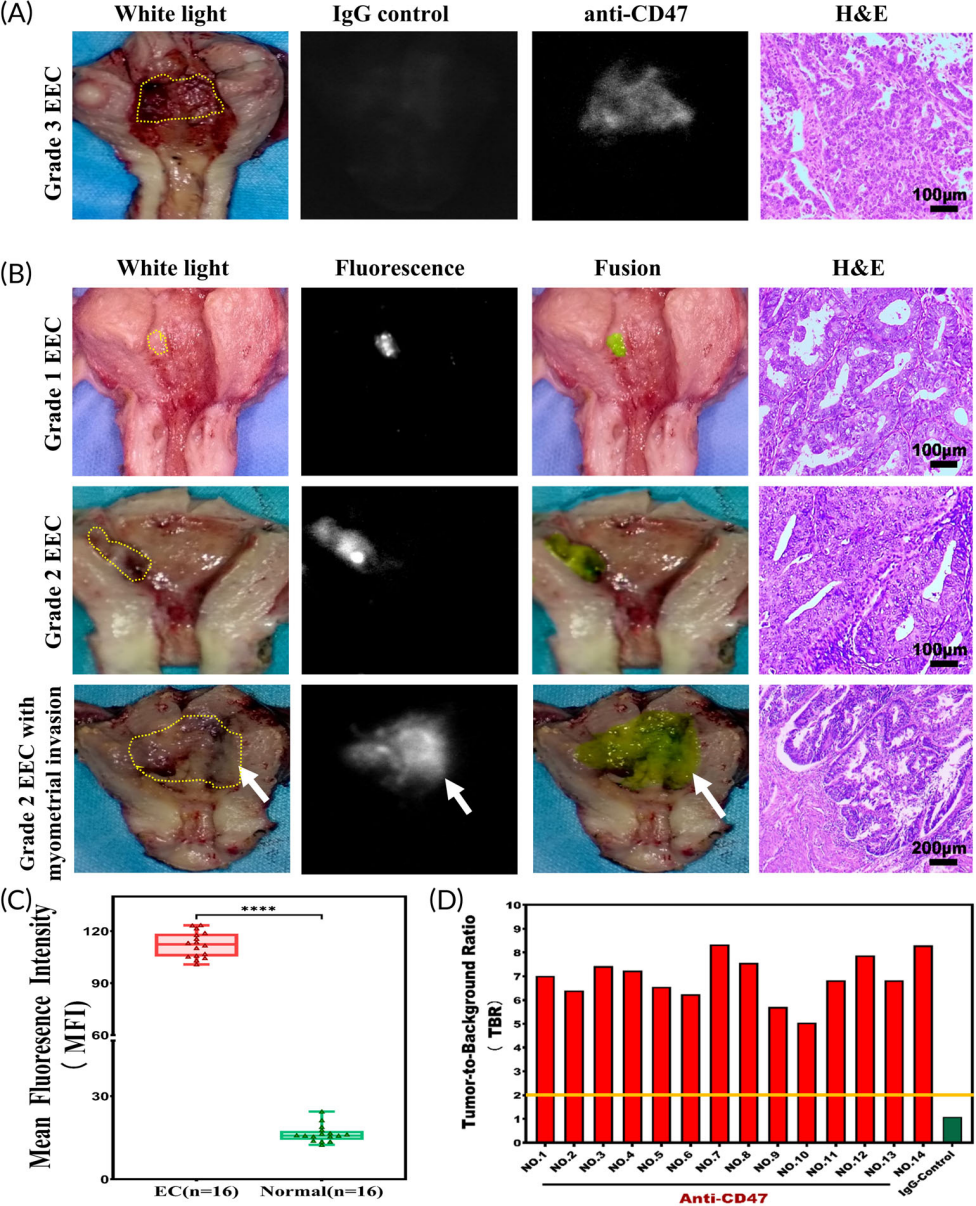
NIR molecular imaging of freshly isolated human endometrial images with the relevant hematoxylin and eosin‐stained (H&E, ×200) photomicrographs for colocalization of CD47‐AF790 binding and histopathology. (A) One EC specimen was incubated and imaged successively with IgG control‐Alexa Fluor 790 and CD47‐AF790. White and NIR‐light images of EC after incubation with the two antibodies were shown. (B) Representative images of EC after incubation with CD47‐AF790. Positive fluorescence was observed in the uterine cavity where the cancer invaded the myometrium (at the arrow). (C) Quantitative MFI for 16 samples. The MFIs of tumor tissue and normal tissue were 112.22 ± 7.38 versus 16.39 ± 3.04, respectively. (D) Tumor‐to‐background ratio (TBR) data for each patient.

In the study, CD47‐targeted NIR molecular imaging was performed on 16 ex vivo samples (Figure 7). To evaluate the specificity and feasibility of CD47 as an optical molecular target in EC imaging, we performed NIR imaging on fresh ex vivo human EC tissues. Preliminary experiments were designed to ascertain whether the fluorescence observed in cancerous tissues bound specifically to CD47. Initially, a sample was treated with isotype immunoglobulin G (IgG)‐Alexa Fluor 790 as a negative control. As a result, only faint fluorescence was observed in both cancerous and normal endometrial tissues (MFI 16.14 ± 3.18 and 15.57 ± 4.50, respectively). Subsequently, the same tissue sample was treated with CD47‐AF790, thoroughly washed with sterile saline, and reimaged. Under NIR light, the fluorescence intensity in the cancerous areas significantly increased, showing a 7.02‐fold enhancement, while the fluorescence intensity in normal tissues remained relatively unchanged (MFIs of 110.28 ± 6.49 and 15.71 ± 3.90, respectively, Figure 8A).

Figure 8B illustrates notable imaging results from tumors in the uterine cavity, highlighting the improved contrast and visual identification of tumors through OMI technology. To summarize, the quantitative evaluation of fluorescence signals across all patients revealed a notable difference in MFI between tumor tissues and the adjacent normal tissues when utilizing NIR OMI techniques (112.22 ± 7.38 vs. 16.39 ± 3.04, *p* < 0.0001, Figure 8C). Histopathological examination revealed that regions with elevated fluorescence correlated with endometrioid adenocarcinoma, while non‐fluorescent areas indicated normal tissue. Notably, in Figure 8B the arrow indicated significant fluorescence in the myometrium, which was later identified as lesion invasion through pathology. The study concluded that the TBR of tumor lesions in freshly excised whole uterine specimens ranged from 5.05 to 8.34 (Figure 8D).

**FIGURE 9:**
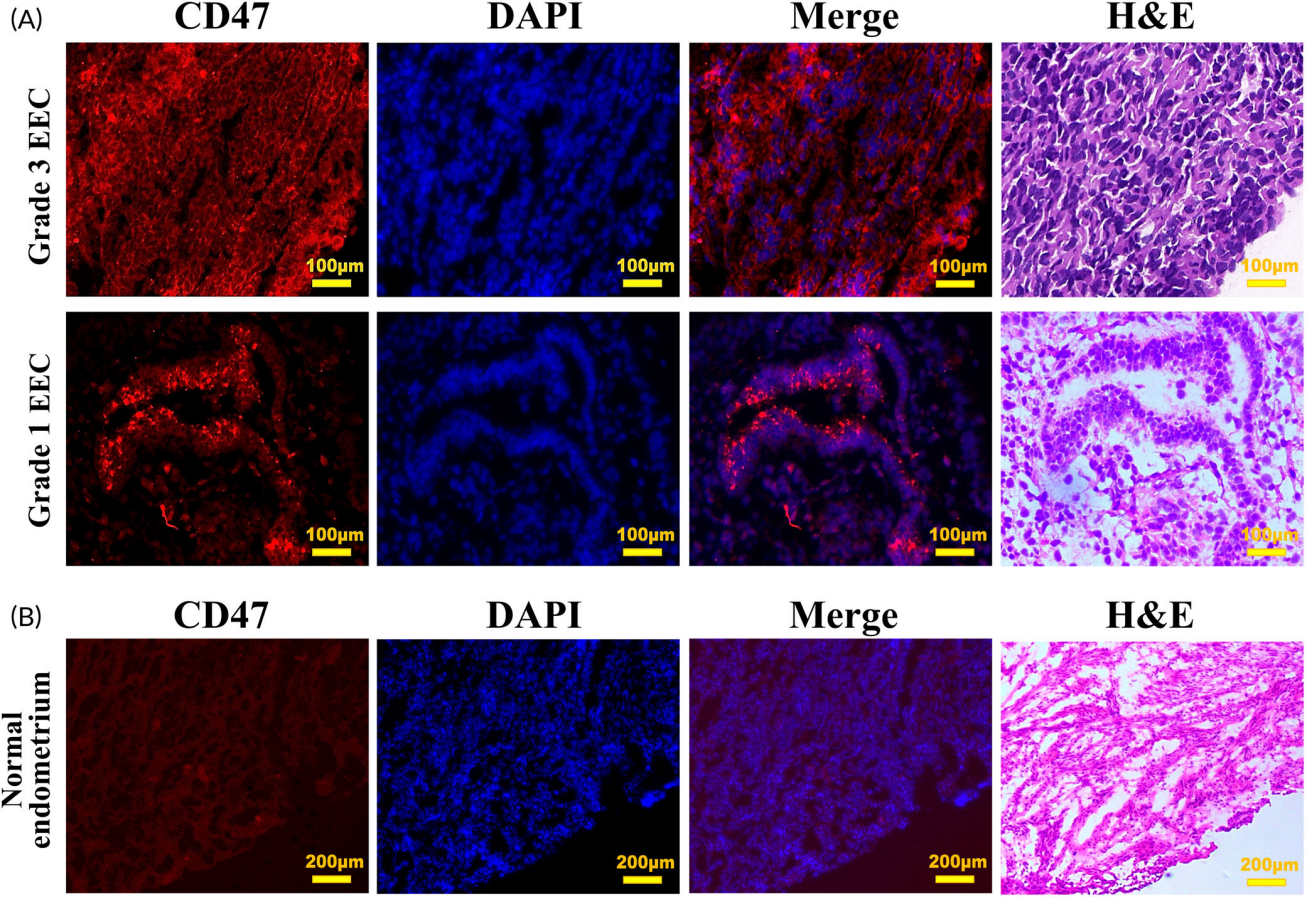
Histopathological evaluation of CD47 fluorescence signals in EC and paracancerous tissue. (A) Representative images under fluorescence microscopy showed the accumulation of CD47 on the membrane of tumor cells. The fluorescence signal was represented in red, and cell nuclei stained with DAPI were shown in blue. Comparative analysis with hematoxylin and eosin (H&E) staining on the same horizontal histological section confirmed that the fluorescence distribution represented tumor lesions. (B) No CD47‐specific binding fluorescence was observed in the paracancerous tissue.

To further validate the specific binding and distribution of CD47 in EC tissues, we analyzed a specimen by preparing frozen sections of both the fluorescent tumor lesions and the surrounding non‐cancerous tissue. These sections were then subjected to H&E staining at the same level. The results revealed that under fluorescence microscopy, specific fluorescence signals were observed on tumor cells, and CD47 was predominantly located on the membrane of these tumor cells (Figure 9A), whereas no significant fluorescent signals were seen on the normal endometrial tissues adjacent to the cancer (Figure 9B). Histopathology further confirmed the consistency between fluorescence and tumor presence. Therefore, this investigation suggests employing CD47 alongside OMI technology to precisely locate and remove lesions intraoperatively based on the TBR, offering an innovative method for EC therapeutic strategies with a focus on fertility preservation.

## 4 DISCUSSION

Hysteroscopic surgery is an increasingly recognized approach for the evaluation and removal of tumor lesions in the fertile‐preserving treatment of early‐stage EC.^17^ Nonetheless, its subjective nature and limited accuracy in diagnosing conditions such as endometrial hyperplasia can hinder its effectiveness in identifying tumor lesions.^18^ A study showed that hysteroscopic endometrial biopsy misdiagnosed EC as endometrial hyperplasia in some cases, thereby underscoring the difficulty in distinguishing between early‐stage tumors and hyperplasia, resulting in a sensitivity of 84.6% and a negative predictive value of 87.5% for hysteroscopic examinations.^19^ Additionally, a meta‐analysis also indicated the high accuracy of hysteroscopy in diagnosing EC but its limitations in identifying non‐specific endometrial conditions like hyperplasia.^20^

In light of these diagnostic challenges, the integration of advanced optical imaging technologies with hysteroscopy is becoming increasingly prevalent. Currently, techniques used for lesion identification during hysteroscopy include white‐light hysteroscopy, narrow‐band imaging (NBI), photodynamic diagnosis (PDD), confocal laser endoscopy (CLE), etc. However, each of these approaches has its specific constraints and drawbacks. For example, while PDD and NBI can improve the detection of precancerous lesions, their lack of tumor specificity often results in a high rate of false positives in conditions like atrophic or hyperplastic endometrium, hemorrhage and inflammation.^21^, ^22^ CLE, offering rapid and detailed cellular‐level lesion diagnosis, is restricted by its limited field of view and often requires combination with broader imaging techniques.^23^ Optoacoustic (OA) imaging is also a new non‐invasive and radiation‐free imaging method that has been rapidly developed in recent years. It combines the high spatial resolution of ultrasound with the high contrast of optical imaging to produce highly specific tissue images.^24^ However, it faces several challenges: improving the imaging quality of acoustically obstructed tissues (e.g., bone and air‐filled cavities), resolving the limited imaging depth,^25^ enhancing image contrast and detection sensitivity,^26^ and developing high‐performance, low‐cost real‐time imaging and dynamic monitoring techniques.^27^

Optical imaging, a rapidly evolving field, allows for the visualization of molecular structures and biological processes with exceptional spatial and temporal resolution.^28^ In particular, OMI significantly enhanced the detection of malignant lesions using targeted molecular tracers that bind specifically to tumor sites.^29^ This technology not only provides real‐time and dynamic imaging during surgery but also assists in pinpointing small, ambiguous lesions, thereby facilitating thorough lesion removal and minimizing the likelihood of post‐operative recurrence.^30^ Consequently, OMI supports surgeons in accurately delineating tumor boundaries during procedures, effectively lowering the rates of positive surgical margins.^31^ Therefore, this study aimed to explore the potential application value of OMI in guiding surgery for fertility preservation in EC.

CD47 belongs to the immunoglobulin superfamily and plays a crucial role as an innate immune checkpoint. Its receptor, signal regulatory protein alpha (SIRPα), predominantly resides on the surface of macrophages. The binding of CD47 to SIRPα on macrophages sends a “don't eat me” signal, thereby suppressing the phagocytic activity of these immune cells and allowing tumor cells to evade immune response.^32^ In various malignant tumors, inhibiting the interaction between CD47 and SIRPα has been demonstrated to boost the phagocytosis of cancer cells by macrophages, promoting their clearance.^13^ This enables CD47 to become an important biomarker for cancer treatment and prognosis. Our previous research has validated the potential of utilizing CD47 as a target for OMI in bladder cancer and upper urinary tract urothelial carcinoma. This approach has proven effective in increasing the detection rates of early and small tumors and improving tumor visualization.^33^, ^34^ Recent studies have highlighted the high expression of CD47 in EC.^35^, ^36^

However, compared to antibodies and antibody fragments, peptides and small molecules are smaller, easier to produce, and more cost‐effective. There is no research on the differences in the ability of various molecular tracers to recognize EC cells. A literature search on PubMed revealed that Gonadotropin‐Releasing Hormone (GnRH), a hypothalamic decapeptide, is expressed in approximately 50%–95% of human EC. Numerous GnRH analogs have been developed and conjugated with cytotoxic molecules for targeted therapy against cancer cells.^37^, ^38^ Additionally, a study found that the OTL‐38 targeting FRα NIR imaging agent can clearly identify all omental and lymph node metastases in high‐risk EC patients.^39^ Therefore, CD47 antibodies, GnRHa, and folate were selected as targeted imaging molecules for human EC in this study. After incubating EC cells with different targeting molecules, flow cytometry results showed that the affinity between the CD47 antibody and tumor cells was significantly higher than the other two targeting molecules (GnRHa and folate) (the content and figure in the supplement). Thus, we chose the CD47 antibody with the highest affinity for EC.

In this study, we confirmed the feasibility of CD47 antibody as the target for OMI in the diagnosis and treatment of EC. Consistent with previous studies, we demonstrated that CD47 was overexpressed in EC through bioinformatics, immunohistochemistry, and qRT‐PCR. Additionally, flow cytometry revealed that the affinity of CD47 to EC cells was significantly higher than that to normal endometrial epithelial cells. Moreover, the cell immunofluorescence showed that CD47 is primarily distributed on the tumor cell membrane. In summary, we selected CD47 as the molecular imaging target for the treatment of EC aimed at preserving fertility.

Near‐infrared photoimmunotherapy (NIR‐PIT) represents an innovative approach to cancer treatment, targeting cancer cells at the molecular level. This technique involves conjugating antibodies, which target cancer cell surface markers, with hydrophilic photosensitizers. When exposed to NIR light, NIR‐PIT effectively eliminates cancer cells while sparing normal ones.^40^ The use of monoclonal antibodies in NIR‐PIT ensures precise targeting of cancer cells, especially those expressing higher levels of cancer‐related antigens. Once activated by light, NIR‐PIT destroys these cancer cells without harming normal cells, including immune cells in the tumor.^10^ NIR light's non‐ionizing nature prevents DNA or cell damage, enabling the light to penetrate deeper into tissues.^40^ Its selective cell‐killing property makes it suitable for treating residual or recurrent tumors.^41^ Due to its selective action, its precise targeting makes NIR‐PIT a promising option for extensive use in oncology. Currently, NIR‐PIT is clinically applied in treating head and neck squamous cell carcinoma and has shown potential in preclinical studies for treating urological cancers, including bladder and prostate cancers. We demonstrate the feasibility of CD47‐targeted NIR‐PIT in the treatment of EC cells. In EC cell lines, CD47‐targeted NIR‐PIT induced cytotoxicity in a light dose‐dependent manner. Additionally, laser confocal microscopy revealed that CD47 antibody intervention significantly increased the phagocytic effect of macrophages on EC cells. NIR‐PIT mediated by anti‐CD47‐AF790 exhibited dual functions of directly killing effect and enhancing phagocytosis on EC cells. In this study, we established a mouse model of partial or complete tumor resection mediated by CD47‐targeted OMI. In the partial tumor resection model, compared to group A (immune therapy alone), group C (NIR‐PIT treatment) mice showed a reduced tumor recurrence rate after NIR‐PIT intervention. However, the difference did not reach statistical significance. This may be due to the relatively small sample size and variability in tumor growth. In the complete tumor resection model, no tumor recurrence was observed postoperatively, indicating that CD47‐targeted OMI can improve tumor detection. We then evaluated the effect of CD47‐targeted NIR‐PIT maintenance therapy on tumor recurrence in mice. The results indicated that, compared to untreated animals, the tumor growth rate was slower in the NIR‐PIT group using CD47‐AF790, allowing for more sustained tumor control. Therefore, the combination of targeted OMI and NIR‐PIT could achieve targeted therapy, and specifically eliminate exfoliated cells and residual lesions, providing a complementary option for optimizing tumor treatment.

Then we utilized anti‐CD47‐Alexa Fluor790 as a molecular targeting tracer, combining the CD47 antibody with Alexa Fluor790, which is activated by NIR light. The Alexa dyes and their conjugates can exhibit stronger fluorescence intensity and greater photostability compared to traditional dyes like fluorescein, rhodamine, and Cy3.^42^ In this study, incubating CD47‐AF790 molecular fluorescence tracers in freshly isolated specimens from 16 cases of EC, significant differences in fluorescence intensity between EC lesions and normal endometrial epithelium were observed under NIR light (112.22 ± 7.38 vs. 16.39 ± 3.04, respectively). This assisted in the real‐time diagnosis of tumors. Although fertility‐preserving treatment for young EC was indicated for patients with stage IA, G1‐differentiated, noninvasive endometrioid adenocarcinoma,^1^ our study included EC of various grades and stages. We found that CD47‐AF790 demonstrated high specificity and sensitivity in targeting EC. Additionally, we validated the specific attachment of CD47 to EC tissues through tissue immunofluorescence. This study laid the foundation for further exploration of the targeting specificity of CD47 in EC, as well as the feasibility and subsequent advancements in this methodology. This research advocates for the integration of CD47 with OMI technology for precise intraoperative localization and excision of lesions based on the TBR, introducing a novel avenue for EC treatment options, particularly those aimed at conserving fertility.

This study must emphasize certain limitations. Firstly, the number of patients in this ex vivo feasibility study was small, and the patients included were selectively chosen. The current findings still require further validation in larger cohorts. However, we believe that these findings are highly insightful for gynecologists and patients seeking fertility preservation. Gynecologists can accurately identify and excise lesions during surgery, preserve normal tissue and reduce the risk of recurrence, thereby offering the possibility of retaining fertility function. Secondly, this study did not incorporate the molecular subtyping of EC into the selection of patients suitable for fertility‐preserving treatments. As research into the molecular characteristics of tumors deepens, the Cancer Genome Atlas (TCGA) has categorized EC into four types: POLE mutant, high microsatellite instability (MSI‐H)/dMMR, low copy number/TP53 wild‐type/no specific molecular profile (NSMP), and high copy number (CNH)/TP53 mutant.^43^ The significance of molecular subtyping for fertility‐preserving treatment in EC lies in its potential to indicate prognosis and predict the response to progestin. However, due to the limited number of cases currently included in the study and ongoing clinical research exploring precise assessments for determining the suitability of patients for fertility‐preserving treatments, the application of molecular subtyping in such treatments lacks substantial data support. In the future, it is hoped that the study group will make optimal choices for indications and plans of fertility‐preserving treatments in larger cohorts based on molecular characteristics. Lastly, for clinical translation, the establishment of human‐applicable targeted tracers is needed. The CD47 antibody B6H12 we used is a monoclonal antibody, which may not be safe for human use. However, the humanized monoclonal CD47 antibody Hu5F9‐G4 is currently undergoing a phase I clinical trial in patients with advanced solid malignancies or lymphomas, and the results indicate that most toxic reactions are mild to moderate.^44^ These involved transient anemia (57%), fatigue (64%), headaches (50%), and so on.^44^ The clinical translation of OMI ultimately requires interdisciplinary collaboration among clinical doctors, chemists, physicists, pharmacologists, and engineers, each bringing their unique expertise to ensure the translation of these findings into effective, practical and safe clinical applications.

In summary, CD47‐targeted molecular fluorescent tracers incubated with freshly isolated uterine specimens, and NIR light images revealed that high MFI signals could be used as a predictive factor for residual lesions. This allows gynecologists to make real‐time assessments of positive margins during surgery. Thus, our study describes a significant advancement in medical imaging technology, in which the integration of CD47 with OMI technology markedly enhances image contrast. This enhanced image contrast significantly helps the surgeons to precisely identify lesions and achieve complete surgical resection during surgery, while preserving normal tissue and minimizing surgical complications.

## 5 CONCLUSIONS

In our study, we have demonstrated the feasibility of CD47 as an optical molecular target for EC. CD47‐targeted NIR‐PIT has a dual effect on killing cancer cells directly and enhancing phagocytosis in a light dose‐dependent manner. CD47‐AF790 molecular fluorescent tracer, combined with freshly isolated whole uterine specimens, significantly enhanced the contrast of NIR visual images of tumor lesions. Combining OMI with NIR‐PIT can improve the accurate identification of tumor tissue, while PIT can target and eliminate residual lesions and shed tumor cells, thus realizing the need for integrated tumor diagnosis and treatment. Therefore, NIR molecular imaging targeting CD47 in this study has proved to be a promising method for diagnosis and treatment. The results provide a theoretical basis for the application of OMI in preserving fertility in early‐stage EC patients and offer new insights for broader diagnostic and therapeutic strategies.

## AUTHOR CONTRIBUTIONS

**Jing Lei:** Conceptualization; methodology; validation; data curation; writing – original draft; writing – review and editing. **Dianfeng Tian:** Methodology; software; validation; writing – original draft. **Bo Zhang:** Methodology; validation; writing – review and editing. **Hongrui Guo:** Data curation; writing – original draft; writing – review and editing. **Huancheng Su:** Validation; formal analysis. **Jinzheng Wei:** Methodology. **Shuai Li:** Software; data curation. **Sufen Li:** Visualization. **Chao Liu:** Writing – review and editing. **Xiaofeng Yang:** Conceptualization; supervision; funding acquisition. **Sanyuan Zhang:** Project administration.

## CONFLICT OF INTEREST STATEMENT

The authors declare no conflict of interest.

## Supporting information


**Appendix S1:** Supporting information.

## Data Availability

The data that supports the findings of this study are available in the supplementary material of this article.

## References

[1] Abu‐Rustum N , Yashar C , Arend R , et al. Uterine neoplasms, version 1.2023, NCCN clinical practice guidelines in oncology. JNCCN. 2023;21(2):181‐209. 36791750 10.6004/jnccn.2023.0006

[2] Crosbie E , Kitson S , McAlpine J , Mukhopadhyay A , Powell M , Singh N . Endometrial cancer. Lancet. 2022;399(10333):1412‐1428. 35397864 10.1016/S0140-6736(22)00323-3

[3] Matsuo K , Mandelbaum R , Matsuzaki S , Klar M , Roman L , Wright J . Ovarian conservation for young women with early‐stage, low‐grade endometrial cancer: a 2‐step schema. Am J Obstet Gynecol. 2021;224(6):574‐584. 33412129 10.1016/j.ajog.2020.12.1213

[4] Olga VN , Vladimir BN , Vladimir AP , et al. Live births and maintenance with levonorgestrel IUD improve disease‐free survival after fertility‐sparing treatment of atypical hyperplasia and early endometrial cancer. Gynecol Oncol. 2021;161(1):152‐159. 33461741 10.1016/j.ygyno.2021.01.001

[5] Jeong‐Yeol P , Joo‐Hyun N . Progestins in the fertility‐sparing treatment and retreatment of patients with primary and recurrent endometrial cancer. Oncologist. 2015;20(3):270‐278. doi:10.1634/theoncologist.2013-0445 25673106 PMC4350794

[6] Park J , Kim D , Kim J , et al. Long‐term oncologic outcomes after fertility‐sparing management using oral progestin for young women with endometrial cancer (KGOG 2002). Eur J Cancer. 2013;49(4):868‐874. 23072814 10.1016/j.ejca.2012.09.017

[7] Yang B , Xu Y , Zhu Q , et al. Treatment efficiency of comprehensive hysteroscopic evaluation and lesion resection combined with progestin therapy in young women with endometrial atypical hyperplasia and endometrial cancer. Gynecol Oncol. 2019;153(1):55‐62. 30674421 10.1016/j.ygyno.2019.01.014

[8] Mazzon I , Corrado G , Masciullo V , Morricone D , Ferrandina G , Scambia G . Conservative surgical management of stage IA endometrial carcinoma for fertility preservation. Fertil Steril. 2010;93(4):1286‐1289. 19700153 10.1016/j.fertnstert.2008.12.009

[9] Lauwerends L , van Driel P , Baatenburg de Jong R , et al. Real‐time fluorescence imaging in intraoperative decision making for cancer surgery. Lancet Oncol. 2021;22(5):e186‐e195. 33765422 10.1016/S1470-2045(20)30600-8

[10] Kobayashi H , Choyke P . Near‐infrared photoimmunotherapy of cancer. Acc Chem Res. 2019;52(8):2332‐2339. 31335117 10.1021/acs.accounts.9b00273 PMC6704485

[11] Mohiuddin T , Zhang C , Sheng W , et al. Near infrared photoimmunotherapy: a review of recent progress and their target molecules for cancer therapy. Int J Mol Sci. 2023;24(3):2655. doi:10.3390/ijms24032655 36768976 PMC9916513

[12] van Duijn A , Van der Burg S , Scheeren F . CD47/SIRPα axis: bridging innate and adaptive immunity. J Immunother Cancer. 2022;10(7):e004589. doi:10.1136/jitc-2022-004589 35831032 PMC9280883

[13] Narla R , Modi H , Bauer D , et al. Modulation of CD47‐SIRPα innate immune checkpoint axis with fc‐function detuned anti‐CD47 therapeutic antibody. Cancer Immunol Immunother. 2022;71(2):473‐489. 34247273 10.1007/s00262-021-03010-6 PMC10992396

[14] Yang M , Jiang C , Li L , Xing H , Hong L . Expression of CD47 in endometrial cancer and its clinicopathological significance. J Oncol. 2022;2022:7188972. 35281519 10.1155/2022/7188972 PMC8916881

[15] Sercan Ç , Haberal Reyhan A , Özen Ö , Ayhan A . Clinicopathologic and prognostic significance of CD47 expression and tumor‐associated macrophages in endometrial carcinoma. Int J Gynecol Pathol. 2022;41(4):397‐406. 34282107 10.1097/PGP.0000000000000809

[16] Jensen MM , Jørgensen JT , Binderup T , Kjaer A . Tumor volume in subcutaneous mouse xenografts measured by microCT is more accurate and reproducible than determined by 18F‐FDG‐microPET or external caliper. BMC Med Imaging. 2008;8:16. 18925932 10.1186/1471-2342-8-16 PMC2575188

[17] Rodolakis A , Pergialiotis V , Thomakos N . New boundaries for fertility sparing management in endometrial cancer. Curr Opin Oncol. 2023;35(5):394‐400. 37498119 10.1097/CCO.0000000000000974

[18] Gkrozou F , Dimakopoulos G , Vrekoussis T , et al. Hysteroscopy in women with abnormal uterine bleeding: a meta‐analysis on four major endometrial pathologies. Arch Gynecol Obstet. 2015;291(6):1347‐1354. 25524536 10.1007/s00404-014-3585-x

[19] Garuti G , Mirra M , Luerti M . Hysteroscopic view in atypical endometrial hyperplasias: a correlation with pathologic findings on hysterectomy specimens. J Minim Invasive Gynecol. 2006;13(4):325‐330. 16825075 10.1016/j.jmig.2006.03.010

[20] Clark T , Voit D , Gupta J , Hyde C , Song F , Khan K . Accuracy of hysteroscopy in the diagnosis of endometrial cancer and hyperplasia: a systematic quantitative review. Jama. 2002;288(13):1610‐1621. 12350192 10.1001/jama.288.13.1610

[21] Peitsidis P , Vrachnis N , Sifakis S , et al. Improving tissue characterization, differentiation and diagnosis in gynecology with the narrow‐band imaging technique: a systematic review. Exp Ther Med. 2022;23(1):36. 34849151 10.3892/etm.2021.10958 PMC8613536

[22] Matoba Y , Banno K , Kisu I , et al. Hysteroscopic photodynamic diagnosis using 5‐aminolevulinic acid: a high‐sensitivity diagnostic method for uterine endometrial malignant diseases. J Minim Invasive Gynecol. 2020;27(5):1087‐1094. 31415818 10.1016/j.jmig.2019.08.012

[23] Wen J , Yang X , Ye G , Chen R , Feng Y , Liao Q . Preliminary study of confocal laser endomicroscopy for in vitro specimens of the endometrium. BMC Cancer. 2022;22(1):1094. 36284282 10.1186/s12885-022-10137-x PMC9594941

[24] Kesharwani A , Gujrati V . Multimodal techniques and strategies for chemical and metabolic imaging at the single‐cell level. Curr Opin Biotechnol. 2024;88:103149. 38810301 10.1016/j.copbio.2024.103149

[25] Wen Y , Guo D , Zhang J , et al. Clinical photoacoustic/ultrasound dual‐modal imaging: current status and future trends. Front Physiol. 2022;13(13):1036621. doi:10.3389/fphys.2022.1036621 36388111 PMC9651137

[26] Chen Z , Gezginer I , Zhou Q , Tang L , Deán‐Ben X , Razansky D . Multimodal optoacoustic imaging: methods and contrast materials. Chem Soc Rev. 2024;53(12):6068‐6099. 38738633 10.1039/d3cs00565h PMC11181994

[27] Assi H , Cao R , Castelino M , et al. A review of a strategic roadmapping exercise to advance clinical translation of photoacoustic imaging: from current barriers to future adoption. Photoacoustics. 2023;32:100539. 37600964 10.1016/j.pacs.2023.100539 PMC10432856

[28] Zhang Y , Zhang G , Zeng Z , Pu K . Activatable molecular probes for fluorescence‐guided surgery, endoscopy and tissue biopsy. Chem Soc Rev. 2022;51(2):566‐593. 34928283 10.1039/d1cs00525a

[29] Hernot S , van Manen L , Debie P , Mieog J , Vahrmeijer A . Latest developments in molecular tracers for fluorescence image‐guided cancer surgery. Lancet Oncol. 2019;20(7):e354‐e367. 31267970 10.1016/S1470-2045(19)30317-1

[30] Jiao J , Zhang J , Yang F , et al. Quicker, deeper and stronger imaging: a review of tumor‐targeted, near‐infrared fluorescent dyes for fluorescence guided surgery in the preclinical and clinical stages. Eur J Pharm Biopharm. 2020;152:123‐143. 32437752 10.1016/j.ejpb.2020.05.002

[31] Wojtynek N , Mohs A . Image‐guided tumor surgery: the emerging role of nanotechnology. Wiley Interdiscip Rev Nanomed Nanobiotechnol. 2020;12(4):e1624. 32162485 10.1002/wnan.1624 PMC9469762

[32] B R , vM T , B E . CD47‐SIRPα blocking‐based immunotherapy: current and prospective therapeutic strategies. Clin Transl Med. 2022;12(8):e943. doi:10.1002/ctm2.943 35908284 PMC9339239

[33] Hao H , Wang X , Qin Y , et al. Ex vivo near‐infrared targeted imaging of human bladder carcinoma by ICG‐anti‐CD47. Front Oncol. 2023;13(13):1083553. doi:10.3389/fonc.2023.1083553 36937442 PMC10014561

[34] Yan P , Chen D , Yan X , et al. Ex vivo near‐infrared molecular imaging of human upper urinary tract urothelial carcinoma with a CD47‐based targeted tracer. Front Oncol. 2022;12(12):825476. doi:10.3389/fonc.2022.825476 35295998 PMC8919026

[35] Liu Y , Chang Y , He X , et al. CD47 enhances cell viability and migration ability but inhibits apoptosis in endometrial carcinoma cells via the PI3K/Akt/mTOR signaling pathway. Front Oncol. 2020;10:1525. 32984001 10.3389/fonc.2020.01525 PMC7479237

[36] Gu S , Ni T , Wang J , et al. CD47 blockade inhibits tumor progression through promoting phagocytosis of tumor cells by M2 polarized macrophages in endometrial cancer. J Immunol Res. 2018;2018:6156757. 30525058 10.1155/2018/6156757 PMC6247569

[37] Cho‐Clark MJ , Watkins A , Wu TJ . The role of GnRH metabolite, GnRH‐(1‐5), in endometrial cancer. Front Endocrinol Lausanne. 2023;14:1183278. 37124730 10.3389/fendo.2023.1183278 PMC10140499

[38] Emons G , Gründker C . The role of gonadotropin‐releasing hormone (GnRH) in endometrial cancer. Cells. 2021;10(2):292. doi:10.3390/cells10020292 33535622 PMC7912811

[39] Boogerd LSF , Hoogstins CES , Gaarenstroom KN , et al. Folate receptor‐α targeted near‐infrared fluorescence imaging in high‐risk endometrial cancer patients: a tissue microarray and clinical feasibility study. Oncotarget. 2018;9(1):791‐801. 29416655 10.18632/oncotarget.23155 PMC5787511

[40] Railkar R , Agarwal P . Photodynamic therapy in the treatment of bladder cancer: past challenges and current innovations. Eur Urol Focus. 2018;4(4):509‐511. 30145112 10.1016/j.euf.2018.08.005

[41] Okamoto I , Okada T , Tokashiki K , Tsukahara K . Photoimmunotherapy for managing recurrent laryngeal cancer cervical lesions: a case report. Case Rep Oncol. 2022;15(1):34‐39. 35221967 10.1159/000521435 PMC8832182

[42] Panchuk‐Voloshina N , Haugland R , Bishop‐Stewart J , et al. Alexa dyes, a series of new fluorescent dyes that yield exceptionally bright, photostable conjugates. J Histochem Cytochem. 1999;47(9):1179‐1188. 10449539 10.1177/002215549904700910

[43] Kandoth C , Schultz N , Cherniack A , et al. Integrated genomic characterization of endometrial carcinoma. Nature. 2013;497(7447):67‐73. 23636398 10.1038/nature12113 PMC3704730

[44] Sikic B , Lakhani N , Patnaik A , et al. First‐in‐human, first‐in‐class phase I trial of the anti‐CD47 antibody Hu5F9‐G4 in patients with advanced cancers. J Clin Oncol. 2019;37(12):946‐953. 30811285 10.1200/JCO.18.02018 PMC7186585

