# Supplementary material for: Multimodal near‐infrared molecular imaging of ex vivo endometrial carcinoma via CD47‐based targeted tracer
Source: Bioeng Transl Med. 2025 Feb 4;10(3):e10754. doi: 10.1002/btm2.10754 (PMC12079536; doi:10.1002/btm2.10754)
Supplement: Supplementary file 1 — Appendix S1: Supporting information. [file BTM2-10-e10754-s001.docx]

**Method:**

**Flow cytometry:**

EC cells (KLE, Ishikawa, HEC-1-A) were digested with trypsin when both cells had grown to about 80% confluence and then incubated with CD47-FITC、 GnRHa-FITC and folate-FITC for 30 min at 4℃ in the dark. Flow cytometry was performed on the FACSCanto II system (BD Biosciences, USA). The data were analyzed using FlowJo software (version 10.8.1).

**Result:**

**Analysis of Affinity Between Different Targeted Molecules and EC**

To evaluate candidates for optical molecular imaging in EC, we explored the differences in affinity between various targeted molecules, including Anti-CD47、GnRHa and folate. Flow cytometry analysis revealed that, in EC cell lines (KLE, HEC-1-A, and Ishikawa), the affinity of the CD47 antibody for tumor cells was significantly higher compared to the other two targeted molecules (GnRHa and Folate) (Fig. 1).


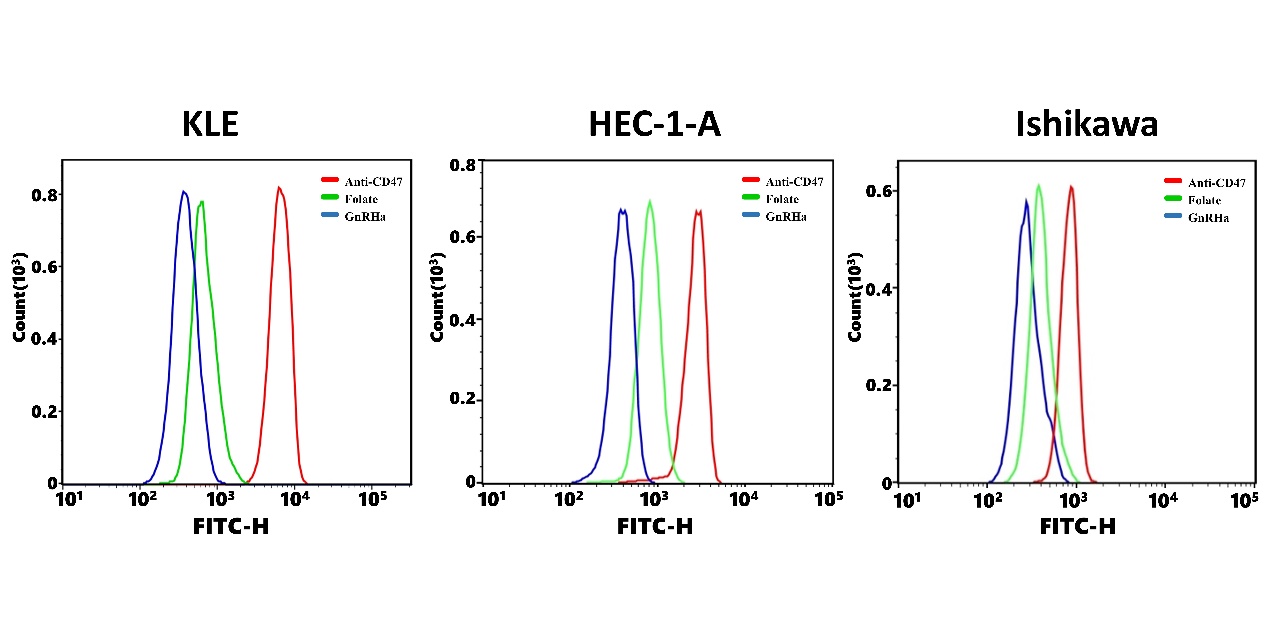
**Fig.1 Affinity between different targeted molecules and EC cell lines**
